# ICP-MS based metallomics and GC-MS based metabolomics reveals the physiological and metabolic responses of *Dendrobium huoshanense* plants exposed to Fe_3_O_4_ nanoparticles

**DOI:** 10.3389/fnut.2022.1013756

**Published:** 2022-09-23

**Authors:** Zhaojian Wang, Jing Wu, Zongping Sun, Weimin Jiang, Yingying Liu, Jun Tang, Xiaoxi Meng, Xinglong Su, Liping Wu, Longhai Wang, Xiaohu Guo, Daiyin Peng, Shihai Xing

**Affiliations:** ^1^College of Pharmacy, Anhui University of Chinese Medicine, Hefei, China; ^2^Anhui Province Key Laboratory of Environmental Hormone and Reproduction, Anhui Province Key Laboratory of Embryo Development and Reproductive Regulation, Fuyang Normal University, Fuyang, China; ^3^Hunan Key Laboratory for Conservation and Utilization of Biological Resources in the Nanyue Mountainous Region, College of Life Sciences and Environment, Hengyang Normal University, Hengyang, China; ^4^College of Humanities and International Education Exchange, Anhui University of Chinese Medicine, Hefei, China; ^5^Department of Horticultural Science, University of Minnesota, Saint Paul, MN, United States; ^6^School of Integrated Chinese and Western Medicine, Anhui University of Chinese Medicine, Hefei, China; ^7^Institute of Traditional Chinese Medicine Resources Protection and Development, Anhui Academy of Chinese Medicine, Hefei, China; ^8^MOE-Anhui Joint Collaborative Innovation Center for Quality Improvement of Anhui Genuine Chinese Medicinal Materials, Hefei, China; ^9^Anhui Province Key Laboratory of Research & Development of Chinese Medicine, Hefei, China

**Keywords:** nanoparticles, *Dendrobium huoshanense*, Fe_3_O_4_ NPs, metabolomics, metallomics, physiological response

## Abstract

It is found that the growth of *Dendrobium huoshanense* was dependent on Fe_3_O_4_, while the bioavailability of plants to ordinary Fe_3_O_4_ was low on the earth. In order to improve the growth, quality and yield of *D. huoshanense*, we used Fe_3_O_4_ NPs (100 or 200 mg/L) that was easily absorbed by plants as nano-fertilizer to hydroponically treat seedlings of *D. huoshanense* for 3 weeks. Fe_3_O_4_ NPs induced not only earlier flowering and increased sugar content and photosynthesis, but also stressed to plants, increased MDA content and related antioxidant enzymes activities. Inductively Coupled Plasma Mass Spectrometry (ICP-MS) revealed that Fe_3_O_4_ NPs caused a significant accumulation of Fe and some other nutrient elements (Mn, Co, B, Mo) in stems of *D. huoshanense*. Metabolomics revealed that the metabolites were reprogrammed in *D. huoshanense* when under Fe_3_O_4_ NPs exposure. Fe_3_O_4_ NPs inhibited antioxidant defense-related pathways, demonstrating that Fe_3_O_4_ NPs have antioxidant capacity to protect *D. huoshanense* from damage. As the first study associating Fe_3_O_4_ NPs with the quality of *D. huoshanense*, it provided vital insights into the molecular mechanisms of how *D. huoshanense* responds to Fe_3_O_4_ NPs, ensuring the reasonable use of Fe_3_O_4_ NPs as nano-fertilizer.

**Graphical Abstract:**
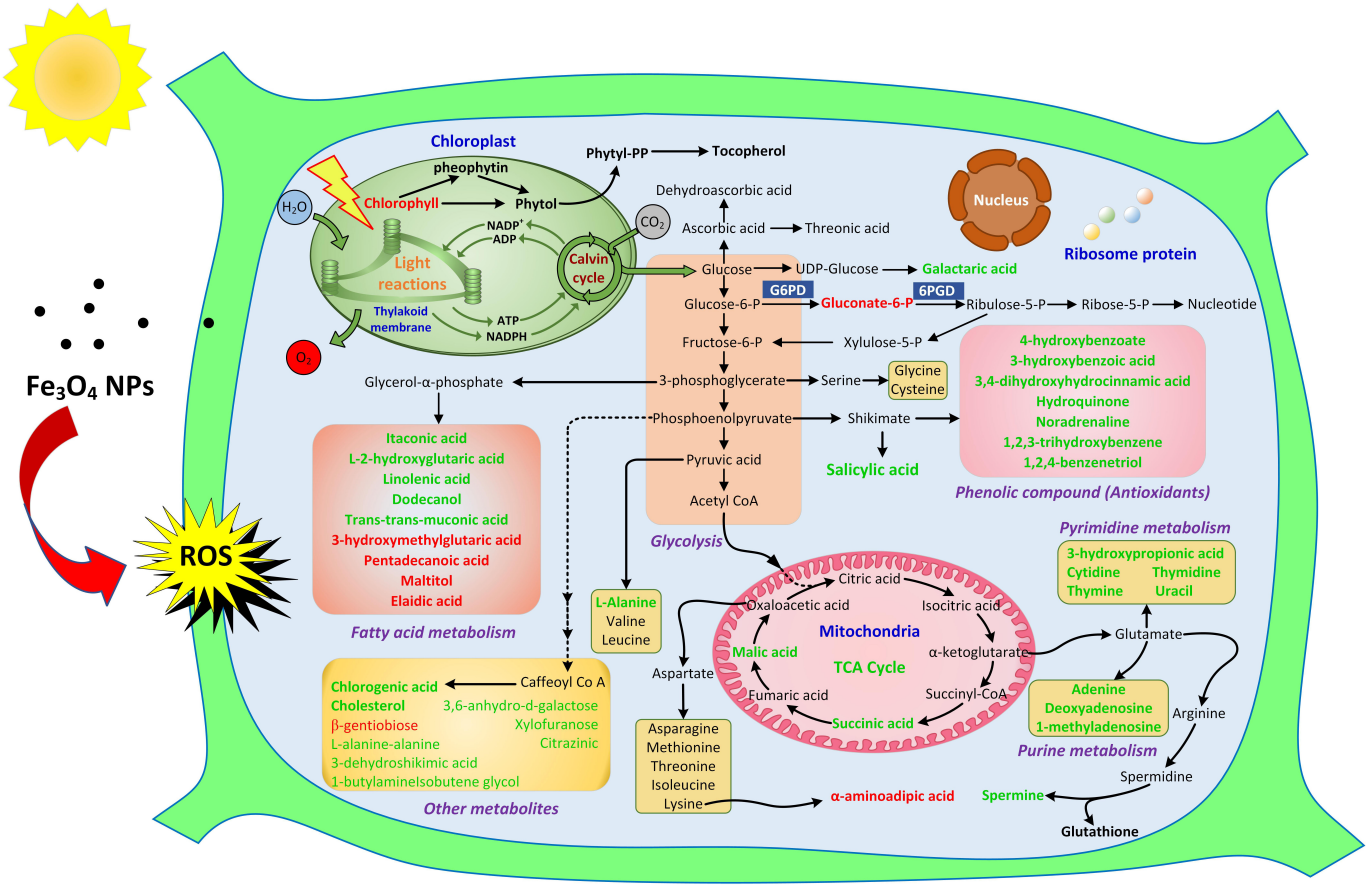


## Introduction

*Dendrobium huoshanense*, a perennial epiphytic herb, is commonly used as a precious high-end medicinal herb and is only found in the mountainous areas of the northern Yangtze River in China. Harboring diverse chemical constituents including polysaccharides, alkaloids, etc. (1), it has several medicinal properties, mainly including hypoglycemic, hypolipidemic, hepatoprotective, immunoregulatory, cataract prevention and tumor proliferation inhibition (2). Wild-type *D. huoshanense* grows on rocks, but better on magnetite rocks than ordinary ones (3). The effects of metallic elements upon *D. huoshanense* have been investigated. It has found Fe^2+^ strongly effected the proliferation (4) and polysaccharide biosynthesis (5) of its protocorm-like bodies. Therefore, there is an urgent need to study the interaction of Fe on plants (6). Although Fe element is enough in the Earth's crust, the Fe^3+^ is not easily absorbed by plants in nature since its insolubility (7).

Nanoparticles (NPs) are extremely tiny, with multiple binding sites and a large surface area, rendering them excellent characteristics as nanocarriers for bioactive molecules (8). The unique photoelectric, physiochemical, and catalytic properties of NPs make them an excellent tool to improve the growth and photosynthesis of plants (9). Accordingly, nanotechnology could be employed to better protect plants and make their production sustainable (10). Even more materials are converted into nanoparticles to promote growth and stress tolerance of plants through foliar spraying, hydroponics or soil route. For example, Shallan et al. (11) used nano-TiO_2_ (50 mg/L) or nano-SiO_2_ (3,200 mg/L) as nanoregulators to enhances drought resistance of cotton (*Gossypium barbadense* L.) plants by foliar spray. Moreover, Ayoub et al. (12) demonstrated the potential entomotoxic effects of CuO NPs and CaO NPs against *Spodoptera littoralis* (cotton leafworm), revealing that nanoengineered metal oxides can be used as cost-effective pesticide formulations. Besides, NPs are more used as nanofertilizers to provide essential nutrients for plants under stress. Subbaiah et al. (13) observed the effects of 50–2,000 ppm ZnO NP (25 nm) on overall growth and zinc transport in maize crops. The results showed that compared with bulk ZnSO_4_, ZnO NPs could significantly increase the germination rate (80%) and seedling vigor index at the concentration of 1,500 ppm. Similarly, Fe_3_O_4_ NPs are smaller to enter plant cells more easily (14), with the released iron is a potential key resource for plants (15).

Many previous studies have illustrated that iron-based NPs have effects on plant growth. Liu et al. (16) cultured seedlings of *Lactuca sativa* with FeO_X_ NPs, which significantly promoted the length of lettuce shoot. The unique peroxidase (POD)-like activity of Fe_3_O_4_ NPs can increase the chlorophyll content and biomass of *Quercus macdougallii* (17) and increase POD enzyme activity of *Cucumis sativus* seedlings (18), respectively. However, iron-based NPs can also have phytotoxic effects on plants. Lee et al. (19) observed that *Arabidopsis* root elongation was inhibited with Fe_3_O_4_ NPs. Ding et al. (20) found that Fe_3_O_4_ NPs significantly hindered the growth of *Eichhornia crassipes*, markedly reducing its chlorophyll content and catalase activity while increasing its malondialdehyde (MDA) content. Therefore, it is paramount that we investigate how to apply Fe_3_O_4_ NPs as nano-fertilizer in the growth of *D. huoshanense*. Considering uncertainty about Fe_3_O_4_ NPs' application, it is primarily necessary to study the metabolite reprogramming of *D. huoshanense* under Fe_3_O_4_ exposure.

As a powerful high-throughput tool, metabolomics can capture and analyze the metabolic status of plants in targeted (21) or untargeted (22), aiming at detecting small molecular metabolites in cells or tissues under specific conditions (23). As the products of gene expression, metabolites can reflect mild variances in gene and protein expression level. Yan et al. (24) treated *Zea mays* with Fe_3_O_4_ NPs and used GC-MS to detect their root metabolites, which revealed the exposure to Fe_3_O_4_ had significant effects on maize root growth and development, as well as cell membrane integrity.

Here, *D. huoshanense* seedlings were grown hydroponically with Fe_3_O_4_ NPs for 3 weeks, and their biochemical parameters, biomass, chlorophyll content, lipid peroxidation, and polysaccharide contents were detected to evaluate the influences of Fe_3_O_4_ NPs. ICP-MS was chosen to determine the content of metal elements. An untargeted metabolomics method using GC-MS was applied to detect the content and composition of metabolites responsing to Fe_3_O_4_ NPs. Our study provides valuable information to improve the quality of *D. huoshanense*.

## Materials and methods

### Plant materials and chemicals

The biennial *D. huoshanense* seedlings, from Huoshan County (Anhui Province, China), were grown at 25 ± 2°C and 23 ± 2°C during the day and night, respectively, under relative humidity at 60–70% and a light/dark cycle at 14 h/10 h in the greenhouse of Anhui University of Chinese Medicine (Hefei, China). Fe_3_O_4_ NPs were bought from the Shanghai Macklin Biochemical Co., Ltd. (Shanghai, China) with 99.5% purity; their original size was ca. 10–30 nm. Standards of (+)-glucose, mannose, glucosamine hydrochloride (>98% purity) were the products of Chengdu Push Bio-technology Co., Ltd. HPLC-grade methanol was bought from Oceanpak. All of other chemicals were analytical grade. Water (HPLC grade) was prepared by a purification system from Pall Filter Co., Ltd. (Beijing, China).

### Experimental design

Uniformly selected *D. huoshanense* seedlings were transferred to a hydroponic system. Every six clusters of seedlings were fixed on a polystyrol-plate in a pot which contained the 1/2 Hoagland solution (25). To prepare Fe_3_O_4_ NPs stock solutions at 100 and 200 mg/L in nanopure water, Fe_3_O_4_ NPs were dissolved in Hoagland solution and sonicated at 45 kHz for 60 min in an ice bath (AS20500BDT, Tianjin Autoscience Instrument Co., Ltd, China), and these applied as treatment groups to *D. huoshanense* seedlings, while the same solution lacking NPs was the control. All seedlings cultivated in a greenhouse for 21 days with their nutrient solution changed every 3 days until harvested. The growth of *D. huoshanense* was observed macroscopically at 0, 7, 14, and 21 days of treatment.

### Biomass and photosynthetic parameter measurements

The seedlings of *D. huoshanense* were treated with their solution for 21 days, and then washed thoroughly with running water for more than 30 min. At last, ultrapure water was used to remove traces of nutrients and Fe ions on their roots' surface by 3 times. After wiping the plants with paper towels, their fresh biomass was measured before oven-drying them (at 65°C for 72 h). Photosynthetic pigment content of seedlings was quantified *in situ*, at the 0, 7, 14, and 21 days (26). The absorbance of total carotenoids and chlorophyll a and b were, respectively, detected at 470, 663, and 645 nm, with a spectrophotometer.

### Malondialdehyde and enzyme crude extract

The crude solutions for Malondialdehyde and the resistance-related enzymes superoxide dismutase (SOD), catalase (CAT), and peroxidase (POD) were extracted as described (27) with several modifications. Leaf samples (each 0.50 g) were ground with an extraction buffer (0.05 M phosphate buffer, pH 7.8) (2 mL) at 4°C, then transferred into centrifuge tube with another 6 mL of extraction buffer. Each mixture was cold centrifuged at 10,000 × g for 20 min. Their supernatants were used to enzyme activities and the MDA content measurement in each treatment group (or control); all measurements were performed in triplicate.

### Lipid peroxidation

MDA content was determined using the 2-thiobarbituric acid (TBA) reaction (28). Two milliliters of the extracted supernatant were mixed with 2 mL of 0.6% TBA, followed by 4 mL of 5% trichloroacetic acid (TCA), mixed and reacted on a boiling water bath for 10 min. The tube was removed to be cooled and centrifuged, and the supernatant was taken to determine its absorbance values at wavelengths of 532, 600, and 450 nm, respectively. The MDA content was expressed as the amount of substance per gram of fresh leaves (μmol/g Fw).

Total SOD activity was quantified by the inhibition of the of nitro blue tetrazolium (NBT)'s photochemical reduction (29). The sample tube reaction system consisted of 1.5 mL 0.05 mol/L phosphate buffer (pH 7.8), 0.3 mL 130 mmol/L methionine (Met), 0.3 mL 0.75 mmol/L NBT, 0.3 mL 0.1 mmol/L EDTA-Na_2_, 0.3 mL 20 μmol/L riboflavin, 0.05 mL enzymatic extract, and 0.25 mL distilled water in a total volume of 3 mL for the reaction mixture. Enzymatic extract in reaction mixture replaced by phosphate buffer was as control tube. Place the sample tube under fluorescent lamp for irradiation (50 μmol/m^2^/s) for 10–20 min (adjust the reaction time appropriately according to the enzyme activity), shade the control tube from light, and record the absorbance at the wavelength of 560 nm. SOD activity was 50% inhibition of NBT reduction by superoxide produced from photo-reduced riboflavin with oxygen. The total SOD activity was expressed in units per gram of fresh leaves (u/g Fw).

The guaiacol method was used for the determination of POD activity (29). A reaction mixture was prepared using 50 mL 0.05 mol/L phosphate buffer (pH 7.8), 28 μL guaiacol, and 19 μL 30% H_2_O_2_ (v/v); 3 mL of the reaction mixture solution was placed into a cuvette with a 1 cm path length. The increase in absorbance at the wavelength of 470 nm was recorded over 4 min at 30 s intervals after the addition of 1 mL enzyme extract. One unit of POD was defined as the amount of enzyme produced a 0.01 absorbance change per minute per gram of fresh leaves at 470 nm [u/(g·min) Fw].

The CAT activity was determined by Gao method (30). A reaction mixture was prepared using 1.5 mL 0.05 mol/L phosphate buffer (pH 7.8), 0.2 mL enzyme extract, and 1 mL ultrapure water; 2.7 mL of the reaction mixture solution was placed into a cuvette with a 1 cm path length. The decrease in absorbance at the wavelength of 240 nm was recorded over 4 min at 30 s intervals after the addition of 0.3 mL 0.1 mol/L H_2_O_2_. One unit of CAT was defined as the amount of enzyme that lowers 0.1 absorbance per minute per gram of fresh leaves at 240 nm [u/(g·min) Fw].

### Iron and other mineral nutrients

The stems of *D. huoshanense* were dried for 72 h at 65°C. From each, a subsample (ca. 0.25 g) of dried tissue was digested by a microwave (Milestone Ethos Up, Italy) in a mixture of 2 mL HNO_3_ and 8 mL H_2_O_2_ (v: v = 1: 4) at 130°C for 10 min and then at 200°C for 30 min. The ultimate solution was diluted to a volume of 50 mL for its analysis. The Fe and other micronutrients (Cu, Mn, Zn, Co, B, Mo, I) contents were determined by ICP-MS (SN02121R, Thermo Fisher Scientific, Germany). Control solution were analyzed between every six samples.

### Bioactive components in *D. huoshanense*

According to the current study, the main bioactive components of *D. huoshanense* are polysaccharides, alkaloids and flavonoids, among which the polysaccharides are mainly composed of glucose and mannose. Using glucosamine as internal standard, the water-soluble polysaccharides was obtained from *D. huoshanense* stems via the hot-water extraction and ethanol precipitation (31). And the monosaccharide solution was obtained by hydrolysis of polysaccharide at high temperature (for 1 h at 110°C). The pre-column derivatization was used by 1-phenyl-3-methyl-5-pyrazolone (PMP) between a given sample solution and standards (mannose and glucose), these then, respectively, washed with chloroform for 2 or 3 times. The supernatant of the water layer was injected for high performance-liquid chromatograph (HPLC) (Agilent 1260 System, Agilent Technologies Inc., CA, USA), to determine each monosaccharide's content.

Following a method described by Wang et al. (32), the contents of total alkaloids and flavonoids in *D. huoshanense* were determined. The former was quantified according to the absorbance value at 620 nm of *Dendrobium* reference substance, the latter by the absorbance value at 510 nm of *Rutin* reference substance.

### Metabolomics analysis

The metabolites of *D. huoshanense* seedlings were analyzed by GC-MS (Agilent Technologies Inc., CA, USA) in the control and treatment groups at their harvest time. Full details of the metabolite isolation and purification, GC-MS based metabolomic analysis, and statistical analysis are given in the following sections.

#### Metabolite extraction

Once harvested, *D. huoshanense* seedlings were thoroughly rinsed to remove any residual particles from their surfaces, and then dried with Kimberly wipes. Next, each fresh whole *D. huoshanense* seedling was ground into powder. From each, accurately weighed 60-mg of subsample and transferred it to a 1.5 mL centrifuge tube containing two small steel balls. The 2-chloro-L-phenylalanine (CAS: 1036) dissolved in methanol (0.3 mg/mL) was set as the internal standard; while the samples were added 40 μL 2-chloro-L-phenylalanine and 360 μL cold methanol and laid aside at −80°C for 2 min, 60 Hz ultrasound for 2 min. All sample tubes were ultrasonicated at room temperature for 30 min, and then chloroform (200 μL) and water (400 μL) were added by another vortex and ultrasonic at room temperature for more than 30 min. Centrifuge each of the above samples at 13,000 rpm for 10 min at 4°C. Samples of quality control were made by mixing all experimental samples in aliquots. Take 100-μL of each supernatant into a glass vial for vacuum-dry at ambient temperature, then add methoxylamine hydrochloride (CAS: 5919) dissolved in pyridine (15 mg/mL, 80 μL). The obtained mixture was vortexized for 2 min and then incubated for 90 min at 37°C, solution of N,O-Bis (trimethylsilyl) trifluoroacetamide (BSTFA) (80 μL) (with 1% trimethylchlorosilane) and n-hexane (20 μL) was then added to the mixture, vigorously vortexed for 2 min and derivatized at 70°C for 60 min. Before GC-MS analysis, all prepared samples were placed at room temperature for 30 min.

#### Metabolomics analysis based on GC-MS

Derived samples after treatment were analyzed using an Agilent 7890B gas chromatography (GC) system (Agilent Technologies Inc., CA, USA), which coupled with a mass selective detector system (Agilent 5977A) and equipped with a fused-silica capillary column in 30 m × 0.25 mm × 0.25 μm size (Model: DB-5MS; Agilent J & W Scientific, Folsom, CA, USA). Helium (>99.999%) was used as the carrier gas through the capillary column at a constant flow rate of 1.2 mL/min. The initial oven temperature was set at 60°C, ramping to 125°C at 8°C/min, 210°C at 5°C/min, 270°C at 10°C/min, 305°C at 20°C/min, and finally maintained at 305°C for 5 min. The 1-μL sample was injected, and the temperature of the injector was set to 300°C in the splitless mode. The temperature of ion source (electron impact) and MS quadrupole was set to 330 and 280°C, respectively. The collision energy was 70 eV, mass data was obtained in a full-scan mode (m/z 50–500), and solvent delay time was set to 5 min. The quality control samples were injected periodically throughout the GC-MS analysis (every 10 samples) to provide an evaluable repeatability data set.

#### Multivariate statistical analysis

In order to visualize the metabolic differences among experimental and control groups, PCA (Principal Component Analysis) and OPLS-DA (Orthogonal Partial Least-Squares-Discriminant Analysis) were performed based on data from GC-MS, via an online analytic method (http://www.metaboanalyst.ca/) (33), following Cao et al. (34). Before analyzed, data were normalized (summation normalization) for differences among samples in general-purpose adjustment, and transformed logarithmically performing to make individual characteristics much more comparable.

Significance of difference among samples was defined as follow: VIP (Variable Importance in Projection) values > 1 in OPLS-DA model analysis, and statistically significant *P*-value (>0.05) from a student's *t*-test (two-tailed) of peak areas in different groups.

### Data processing and statistical analysis

For flowering time and number, chlorophyll, MDA, resistance-related enzyme activity, mineral nutrient contents, and sugar contents were statistically analyzed using one-way ANOVAs (*p* < 0.05) to determine whether mean contents significantly differed among the Fe_3_O_4_ NPs treatments and control. A two-tailed distribution was used to calculate the *p*-values; data are described as mean ± SD (standard error), expressed in three digits.

## Results and discussion

### Properties of Fe_3_O_4_ NPs characterization

The images of the Fe_3_O_4_ NPs from transmission electron microscopy (TEM) were shown in Supplementary Figure 1. The hydrodynamic particle diameter of these particles in ultrapure water at 100 and 200 mg/L, was, respectively, 1002.27 ± 17.66 nm and 1014.33± 18.82 nm, with a corresponding ζ potential of −35.40 ± 0.44 Mv and −46.67 ± 0.40 mV, as detected by an instrument named dynamic light scattering (Zetasizer Nano ZEN3690, Malvern), and pH of 6.86 ± 0.06 and 7.02 ± 0.17, respectively.

### Plant growth and chlorophyll content

Throughout the 21-day growing period, the treated *D. huoshanense* seedlings grew normally and none had any toxicity symptoms. Furthermore, application of Fe_3_O_4_ NPs had no significant impact on their biomass (Supplementary Figure 2A). Interestingly, the Fe_3_O_4_ NPs seemed to unexpectedly affect the flowering of *D. huoshanense* (Supplementary Figure 3). Exposure to 200 mg/L of Fe_3_O_4_ NPs significantly increased the number of flowers of *D. huoshanense*, and both treatments (i.e., 100 and 200 mg/L) induced *D. huoshanense* to flower sooner (Supplementary Figures 2B,C). This early flowering of *D. huoshanense* may be due to oxidative stress caused by exposure to the Fe_3_O_4_ NPs. Stress-induced early flowering is considered a stress-escape response, to ensure species persist, by shortening their life cycle to produce seeds before stress-induced death (35). Similar results were found on peanut seeds treated with ZnO particles in nanoscale: seed germination rate and seedling vigor were significantly greater under the 1,000 ppm nanoscale ZnO treatment, which hastened blossoming and increased the leaf chlorophyll content (36).

It was found that chlorophyll a and b in the 200 mg/L Fe_3_O_4_ NPs group gradually increased in content comparing with the control group, peaking at the 14th day, after which they tended to remain stable. In comparison, the content of chlorophyll in group of 100 mg/L Fe_3_O_4_ NPs stabilize sooner, by 7 days. On the 21st day of harvest, the seedlings' chlorophyll content was significantly higher in the 200 mg/L Fe_3_O_4_ NPs treated group than the control or treating with 100 mg/L Fe_3_O_4_ NPs group (Supplementary Figures 2D,E). Although the content of carotenoids fluctuated during the growth of treated group, it was similar to the control group at the 21st day (Supplementary Figure 2F). These results suggested that Fe_3_O_4_ NPs increased photosynthetic activities and promoted chlorophyll biosynthesis, which was in coordination with many previous studies. Previous study showed that an application of Fe_3_O_4_ NPs and 50 mg/L Fe NPs by spraying on foliar to maize significantly promoted its chlorophyll content about 26.1 and 19.3% over the controls, respectively (37). They speculated that Fe-based NPs might accelerate the electron transport rate within the thylakoid membrane, leading to enhanced photosynthesis. In another report, the superparamagnetic NPs could be transferred into the soybean from the nutrient solution, thereby increasing the chlorophyll content of soybean's sub-apical leaves (38). While in some studies, Fe_3_O_4_ NPs did not affect chlorophyll content in plants. Adding Fe_3_O_4_ NPs to maize grown soil had no impact on the photosynthesis parameters of their leaves as described (24). In our study, cultivating *D. huoshanense* with a Fe_3_O_4_ NPs-containing nutrient solution can also increase their chlorophyll content, which suggests that different Fe-based NPs, different plants and different application modes of NPs can cause different results. Therefore, we need further dissection from physiological and molecular perspectives.

### Biochemical responses of *D. huoshanense*

**Figure 1:**
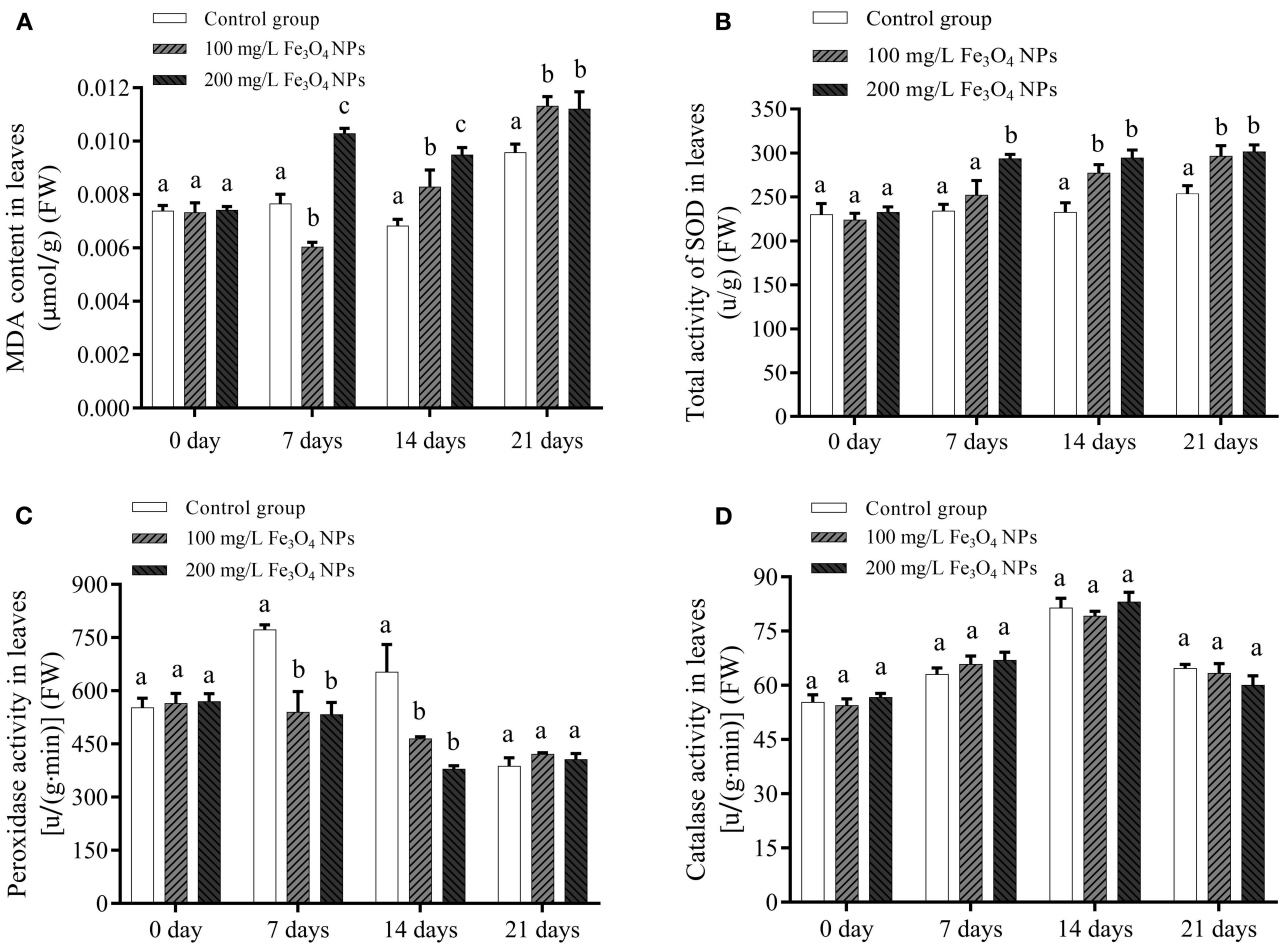
Lipid peroxidation **(A)**, total activity of superoxide dismutase **(B)**, peroxidase activity **(C)** and catalase activity **(D)** of *D. huoshanense* leaves exposed to different doses of Fe_3_O_4_ NPs (0, 100, and 200 mg/L) at different time (0, 7, 14, and 21 day). Data are means of three replicates. FW represents the fresh weight of the samples. Error bars represent standard deviation. Different letters stand for statistical differences at *p* < 0.05.

When plants are subjected to oxidative press, reactive oxygen species (ROS) is formed through the high-energy state electrons transferring to molecular oxygen (O_2_) (39). The toxic effects of ROS can be alleviated by enzymatic and non-enzymatic antioxidant systems. In the former, superoxide dismutase (SOD) is responsible for detoxifying superoxide anions (${\text{O}}_{2}^{-}$), forming H_2_O_2_ and O_2_, of which the toxic chemical H_2_O_2_ can be catabolized by some antioxidant enzymes, such as peroxidase and catalase (40). To test whether different doses of Fe_3_O_4_ NPs induced ROS overproduction and oxidative press, MDA, as a known physiological biomarker of lipid peroxidation in plants, its content in each group was determined. Evidently, MDA content of the control group increased only at the 21st day and did not change before that, whereas in response to 200 mg/L Fe_3_O_4_ NPs it increased significantly at the 7th day and then fluctuated. After being exposed to 100 mg/L Fe_3_O_4_ NPs, the MDA concentration initially fell and then rose continually during the entire cultivation period. The MDA contents of the two treatment groups significantly exceed that of the control group at 21 days (Figure 1A). This indicated that 200 mg/L Fe_3_O_4_ NPs caused oxidative stress to *D. huoshanense* sooner, while oxidative stress was induced by 100 mg/L Fe_3_O_4_ NPs obvious latter. Belonging to metalloenzyme family, SODs can be categorized on the basis of their metal cofactors into 3 groups such as Mn-SOD, Fe-SOD and Cu/Zn-SOD (41). The activity of SOD depends on the presence of the metal co-factor in its vicinity (42). Here, the change in SOD activity roughly mirrored the changed MDA content (Figure 1B). As explanation is that stress augmented SOD activity to eliminate more ROS, to which more Fe and Mn in plants also contributes. Curiously, from the 7th to 14th day, there was less peroxidase activity in treatment group than the control one (Figure 1C). This could be attributed to the Fe_3_O_4_ NPs possessing an intrinsic peroxidase-like activity (43). The reason why peroxidase activity of *D. huoshanense* was lower under exposure to Fe_3_O_4_ NPs was that they can substitute for the plant's natural enzymes in breaking down excess H_2_O_2_. Further, the catalase levels in Fe_3_O_4_ NPs-treated group were similar to the control one (Figure 1D). Compared with the control group, the changes of MDA and antioxidant enzyme activities in *D. huoshanense* during culture in the experimental group showed that Fe_3_O_4_ NPs induced *D. huoshanense* stress, but excessive ROS were decomposed to avoid plant damage after a series of enzyme system reactions. Moreover, 200 mg/L Fe_3_O_4_ NPs caused a more rapid response in *D. huoshanense* than 100 mg/L Fe_3_O_4_ NPs.

### Main bioactive components in *D. huoshanense*

**Figure 2:**
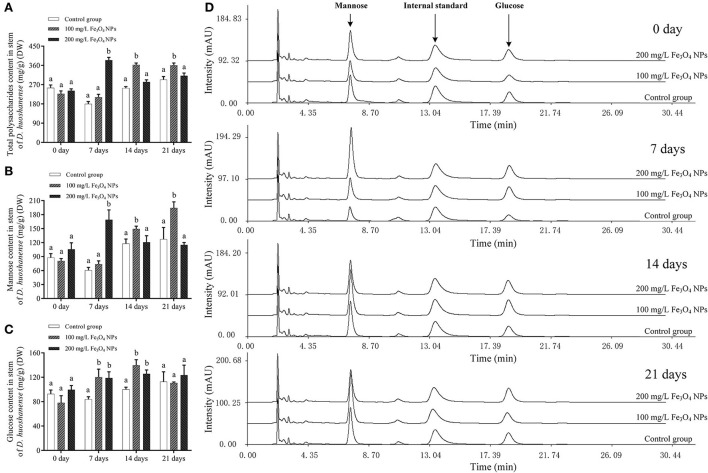
Determination of polysaccharide and monosaccharide contents of *D. huoshanense* cultured with different doses of Fe_3_O_4_ NPs during growth. **(A)** Total polysaccharides contents of *D. huoshanense* samples in different treatment groups at different time. **(B)** Mannose contents of *D. huoshanense* samples in different treatment groups at different time. **(C)** Glucose contents of *D. huoshanense* samples in different treatment groups at different time. **(D)** HPLC-UV chromatogram of *D. huoshanense* samples in different treatment groups at different time. DW represents the dry weight of the samples. Data are means of three replicates. Error bars represent standard deviation. Different letters stand for statistical differences at *p* < 0.05.

In *D. huoshanense*, photosynthesis metabolites, flavonoids, and alkaloids are dominant bioactive metabolites supporting its development and medical efficacy (44). Given that Fe_3_O_4_ NPs increased the chlorophyll content of *D. huoshanense* leaves and enhanced their photosynthesis, the latter would have influenced carbon fixation. To understand how Fe_3_O_4_ NPs exposure impacted carbon fixation in *D. huoshanense*, total contents of polysaccharides and monosaccharides in its stems were determined (Figure 2). The polysaccharide content of *D. huoshanense* peaked at the 7th day, and then declined and stabilized under 200 mg/L Fe_3_O_4_ NPs, that under 100 mg/L Fe_3_O_4_ NPs showed slowly increased after the 7th day and peaked at the 14th day, while that in the control group was slightly increased. The polysaccharides of *D. huoshanense* mainly consisted of mannose and glucose, and the former's trend in variation was roughly consistent with that of polysaccharides. Interestingly, both polysaccharide and mannose content reduced from 7th day to 21st day by 31.98% in the 200 mg/L Fe_3_O_4_ NPs group (Table 1). We speculated the change was caused by the early flowering of *D. huoshanense*, resulting in the excessive consumption of mannose, which also affected the total polysaccharide content. Glucose contents of *D. huoshanense* exposed to Fe_3_O_4_ NPs in concentration of 100 and 200 mg/L were already significantly increased by 7 days, after which they were stable. In the control group, glucose content increased slowly during plant growth and development. It was similar with two treatment groups at 21 days of cultivation. We also found that gene expression in the sugar synthesis pathway in *D. huoshanense* might be influenced by Fe_3_O_4_ NPs, followed by regulating the production of mannose and thus changing the total polysaccharide content in *D. huoshanense*. The changed polysaccharides contents of *D. huoshanense* were consistent with their changed MDA contents. Therefore, we suggest that oxidative stress in *D. huoshanense* is induced by Fe_3_O_4_ NPs exposure, which encouraged the plant to increase its polysaccharide content to alleviate and adapt to the stress. Moreover, the response of plants under 200 mg/L was earlier than that under 100 mg/L Fe_3_O_4_ NPs. This study proved that Fe_3_O_4_ NPs hydroponics could improve the polysaccharides content of *D. huoshanense*, as previously reported (37) that foliar application of Fe_3_O_4_ NPs at 50 mg/L increases the total sugar content in maize roots by 31.1%. Sugar is a key component of carbon flux in most plant species, produced by the fixation of atmospheric carbon via photosynthetic. Increased sugar levels provide a sufficient energy source for cellular respiration to cope with ambient stress. In addition, changes in soluble sugars can help plants to better maintain their water balance and growth (45). Sugars, as carbohydrates, are synthesized in the chloroplasts of leaves and transferred to stems for storage. In our study, Fe_3_O_4_ NPs increased photosynthesis in leaves of *D. huoshanense*, which also explains why Fe_3_O_4_ NPs increased polysaccharide content in stems of *D. huoshanense*. Molecular weight, glycosidic bond and spatial structure of *D. huoshanense* polysaccharide can affect its biological activity. According to the present study (1), the stem polysaccharide of *D. huoshanense* (cDHPS) is composed of → 4) -β-D-glc*p*- (1 → , → 4) -β-D-Man*p*- (1 → , → 4)−3-*O*-acetyl-β-D-Man*p* (1 → , with a molecular weight of 2.59 × 10^5^ Da, Whether Fe_3_O_4_ NPs can affect the structural features of polysaccharides from *D. huoshanense* awaits further investigation.

**Table 1:** Total polysaccharides content and their monosaccharide composition in the stems of *D. huoshanense* (mg/g, FW).

**Sampling time**	**Grouping**	**Total polysaccharides (mg/g)**	**Mannose (mg/g)**	**Glucose (mg/g)**
0 day	Control group	254.6701 ± 14.0599	87.6577 ± 8.7503 a	92.5559 ± 6.5320a
	100 mg/L Fe_3_O_4_ NPs	227.0625 ± 14.0159	80.3854 ± 5.5141 a	77.9835 ± 11.8359a
	200 mg/L Fe_3_O_4_ NPs	241.3811 ± 9.3796	105.4236 ± 14.1549 a	99.5989 ± 6.9097a
7 days	Control group	**179.7537** **±11.5552a**	**60.4167** **±6.3417a**	**83.8290** **±4.2427a**
	100 mg/L Fe_3_O_4_ NPs	**211.0823** **±14.0357a**	**73.5022** **±7.2354a**	**120.0929** **±13.3718b**
	200 mg/L Fe_3_O_4_ NPs	**384.3706** **±13.3575b**	**169.0134** **±21.0616b**	**118.7598** **±10.2131b**
14 days	Control group	**253.6070** **±7.5870a**	**117.7128** **±9.9661a**	**100.0745** **±3.7804a**
	100 mg/L Fe_3_O_4_ NPs	**362.5767** **±8.9461b**	**149.1863** **±6.0745b**	**139.6138** **±9.2311b**
	200 mg/L Fe_3_O_4_ NPs	**272.7432** **±9.8656a**	**120.6365** **±14.0595a**	**125.5985** **±6.4359b**
21 days	Control group	**293.4740** **±14.6336a**	**127.2167** **±25.3721a**	112.6669 ± 16.3714a
	100 mg/L Fe_3_O_4_ NPs	**360.4505** **±11.0713 b**	**194.2425** **±13.1861b**	110.5686 ± 1.7256a
	200 mg/L Fe_3_O_4_ NPs	**261.4146** **±13.2919a**	**114.9530** **±5.2546a**	123.3591 ± 16.6199a

The data are means of three replicates ± standard deviation. Same letters within column indicate no significant difference and different letters stand for significant differences at P < 0.05, highlight the significant differences within the same column by bold values.

The alkaloid and flavonoid contents of *D. huoshanense* in each treatment group were determined as well. Although similar when harvested, differing trends in the content of flavonoids and alkaloids in each treatment group during the cultivation period were discernible (Supplementary Figure 4). Flavonoids have key roles in plant growth and development, such as antibacterial traits, and disease prevention. Few changes in flavonoids further implied that Fe_3_O_4_ NPs rarely affect the normal growth of cultivated *D. huoshanense*.

Alkaloids are a kind of secondary metabolites responsing to habitat stress (46). The total alkaloid content of *D. huoshanense* reached the lowest level at the 7th and 14th day in 100 and 200 mg/L Fe_3_O_4_ NPs treatment groups, respectively. Yet only at the 21st day did the control's alkaloid content did not fall to same level as the Fe_3_O_4_ NPs treatment groups. This may be attributed to the special properties of Fe_3_O_4_ NPs, which protect the plant from oxidative stress, enabling *D. huoshanense* to quickly adjust to its nutrient environment.

### Iron and other minerals

**Figure 3:**
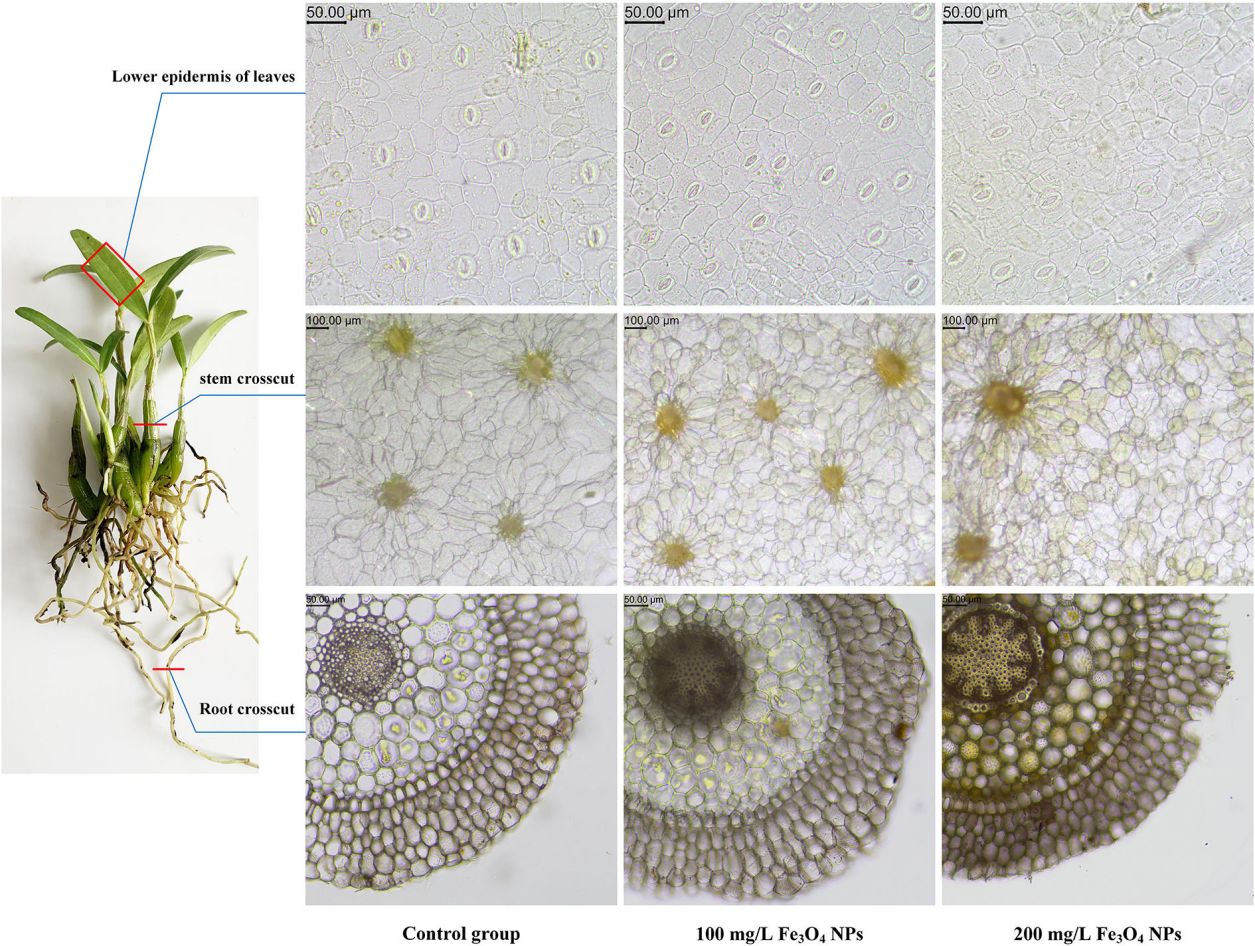
Electron micrograph of roots, stems and leaves sections of *D. huoshanense* treated with different doses of Fe_3_O_4_ NPs (0, 100, and 200 mg/L).

The results suggested that the Fe bioaccumulation in the stem of *D. huoshanense* after 21 days of exposure to NPs was 100.325 ± 7.537, 149.281 ± 4.948, 161.227 ± 10.540 μg/g DW (dried weight) for the 0 (control), 100, and 200 mg/L Fe_3_O_4_ NPs treatment groups, respectively by ICP-MS analysis (Table 2). The cellular changes in roots, stems, and leaves of *D. huoshanense* were observed by electron microscopy at 21 days (Figure 3). With a greater Fe_3_O_4_ NPs treatment concentration, the color of root cells became darker, likely caused by the adsorption of Fe_3_O_4_ NPs near root cortex cells. The stem parenchyma cells of *D. huoshanense* also became smaller and thicker to varying degrees across the Fe_3_O_4_ NPs concentration gradient. Yet among the groups no significant changes were in their leaves' lower epidermal cells, including stomatal density and cell morphology. The Fe content of the control group almost changed before and after treatment. Fe content under the 100 mg/L Fe_3_O_4_ NPs treatment group rose continuously until 14th day and then decreased slightly, as that under the 200 mg/L Fe_3_O_4_ NPs peaked at the 7th day and then declined to a stable level. Fe content in stems of *D. huoshansense* differed between the 100 mg/L and 200 mg/L Fe_3_O_4_ NPs groups. Roots of *D. huoshansense* were directly exposed to Fe_3_O_4_ NPs in different concentrations in nutrient solution during their hydroponic cultivation, we speculated that most of the Fe_3_O_4_ NPs was adsorbed by roots and remained in root epidermis and cortex, however, a portion of Fe_3_O_4_ NPs may enter the phloem, then transported to the stem. Therefore, there are differences in NPs adsorbed and transported into stems by roots of *D. huoshanense* in culture medium containing different concentrations of Fe_3_O_4_ NPs. Furthermore, in this way, Fe_3_O_4_ NPs could influence ROS signaling events from cell to cell, thereby regulating plant metabolism.

**Table 2:** The contents of Fe and other micro-nutrients in *D. huoshanense* stems (μg/g, FW).

**Sampling time**	**Grouping**	**Fe (μg/g)**	**Cu (μg/g)**	**Mn (μg/g)**	**Zn (μg/g)**	**Co (μg/g)**	**B (μg/g)**	**Mo (μg/g)**	**I (μg/g)**
0 day	Control group	99.559 ± 5.911a	3.471 ± 0.738a	22.387 ± 1.940a	33.634 ± 3.742a	0.060 ± 0.029a	5.826 ± 0.199a	0.231 ± 0.039a	0.069 ± 0.006a
	100 mg/L Fe_3_O_4_ NPs	92.880 ± 2.974a	5.131 ± 1.173a	26.093 ± 5.339a	43.543 ± 3.672a	0.050 ± 0.004a	5.586 ± 0.492a	0.254 ± 0.027a	0.076 ± 0.008a
	200 mg/L Fe_3_O_4_ NPs	103.326 ± 6.477a	4.800 ± 0.890a	22.642 ± 5.247a	37.406 ± 4.581a	0.055 ± 0.005a	4.826 ± 0.633a	0.200 ± 0.025a	0.078 ± 0.023a
7 days	Control group	**101.130** **±1.722 a**	**4.699** **±0.962a**	**66.213** **±2.013 a**	**34.765** **±4.723a**	**0.099** **±0.004 a**	**8.187** **±0.290a**	**0.302** **±0.165 a**	2.043 ± 0.042a
	100 mg/L Fe_3_O_4_ NPs	**142.258** **±6.018b**	**3.208** **±0.251b**	**69.582** **±2.427 a**	**44.423** **±1.057b**	**0.099** **±0.010 a**	**6.876** **±0.100b**	**0.414** **±0.010 a**	2.118 ± 0.038a
	200 mg/L Fe_3_O_4_ NPs	**184.428** **±19.209 c**	**4.206** **±0.107b**	**76.563** **±2.152b**	**40.194** **±2.376ab**	**0.139** **±0.011b**	**6.779** **±0.623b**	**0.783** **±0.220b**	2.226 ± 0.206a
14 days	Control group	**111.865** **±6.652 a**	2.199 ± 0.178a	**76.775** **±3.395 a**	43.795 ± 4.952a	**0.155** **±0.018 a**	7.298 ± 0.628a	0.625 ± 0.044a	**2.943** **±0.382ab**
	100 mg/L Fe_3_O_4_ NPs	**159.979** **±3.501b**	3.375 ± 0.685a	**100.178** **±4.073b**	50.205 ± 2.748a	**0.063** **±0.005 a**	8.194 ± 0.068a	0.666 ± 0.047a	**2.524** **±0.190a**
	200 mg/L Fe_3_O_4_ NPs	**155.195** **±33.799b**	3.311 ± 0.501a	**115.231** **±5.643b**	48.533 ± 2.848a	**0.177** **±0.065b**	8.583 ± 0.667a	0.843 ± 0.404a	**3.830** **±0.612b**
21 days	Control group	**100.325** **±7.537 a**	3.257 ± 0.288a	**88.736** **±6.357 a**	41.309 ± 5.028a	**0.107** **±0.001 a**	**7.253** **±1.041a**	**0.344** **±0.013 a**	**1.934** **±0.142a**
	100 mg/L Fe_3_O_4_ NPs	**149.281** **±4.948b**	3.974 ± 0.202a	**114.893** **±8.007 a**	41.880 ± 3.730a	**0.083** **±0.013 a**	**7.739** **±0.355a**	**0.640** **±0.003 a**	**3.192** **±0.546b**
	200 mg/L Fe_3_O_4_ NPs	**161.227** **±10.540 c**	3.706 ± 0.109a	**142.518** **±28.319b**	44.388 ± 2.861a	**0.213** **±0.015b**	**14.657** **±2.460b**	**1.470** **±0.349b**	**5.030** **±0.430c**

The data are means of three replicates ± standard deviation. Same letters within column indicate no significant difference and different letters stand for significant differences at p < 0.05, highlight the significant differences within the same column by bold values.

The ICP-MS results uncovered significantly increased contents of Mn, Co, B, Mo, and I for stems exposed to 100 mg/L and 200 mg/L Fe_3_O_4_ NPs, vis-à-vis the control (Table 2) perhaps because of complementary effects of nutrient elements in *D. huoshanense*. By contrast, the Cu and Zn content were unchanged. In maintaining plant normal growth and development, some micronutrients should be used as cofactors during photosynthesis. Within the plant chloroplasts, 60% to 80% of Mn, Cu, and Fe were found in thylakoids. According to photosynthetic electron transport chain, a total number of 4 Mn atoms, 1 Cu atom, and 22 Fe atoms were required for per chain (41). Therefore, the exposure of plants to the 200 mg/L Fe_3_O_4_ NPs was with higher chlorophyll content than that of control group, which may be caused by their increases of Fe and Mn contents. Cobalt (Co) is the metal component of the cobalamin coenzyme that can promote stem and bud elongation and stabilize protein complex on chloroplast membrane. Boron (B) has structural functions in primary cell walls (47). The above results showed that hydroponic treatment with Fe_3_O_4_ NPs could promote *D. huoshanense* to absorb some metal ions related to plant chloroplasts, and then improve photosynthesis, thereby promoting plant growth and development. However, the mechanism by which Fe_3_O_4_ NPs promote root uptake and transport of corresponding metal ions in *D. huoshanense* remains unclear, and further studies are needed to elucidate how *D. huoshanense* draws nutrients required for growth from rocks.

### Identification of global metabolite profiling

According to the above results for plant physiological indexes and related elements measured in each group, a higher dose of Fe_3_O_4_ NPs could cause greater changes in plants. To explore the influence of a high concentration of Fe_3_O_4_ NPs on *D. huoshanense*, whole plants from the 200 mg/L Fe_3_O_4_ NPs group and control group were selected for GC-MS analysis. Alterations to physiological and biochemical indexes of plants arise from intracellular metabolic changes. A total of 477 metabolites were identified and semi-quantified in *D. huoshanense* by GC-MS. Significantly different compounds were screened after Fe_3_O_4_ NPs treatment (student's *t*-test at *P* < 0.05) through an un-supervised clustering method. PCA was used to draw a visual plot to evaluate, in an unbiased way, the changes in metabolic profiles among the Fe_3_O_4_ NPs groups and the control. The PCA score plots showed that the Fe_3_O_4_ NPs group separated from the control group along the first principal component (PC1) that explained 21% of the total variance (Supplementary Figure 5A). A supervised clustering method of PLS-DA is generally providing a greater discriminative power than a PCA. To gain deeper insight into the metabolite changes, the biased PLS-DA model was applied to the data: the ensuing score plot revealed their clearly separation (Supplementary Figure 5B). The VIP value was used to gauge the explanatory power and influence intensity of each metabolite expression profile on the classification and discrimination of each group's samples. The VIP value represents the overall contribution a given variable to the PLS-DA model, and those metabolites screened at VIP ≥ 1 were the discriminating compounds underpinning differences between groups. Of the 447 metabolites in *D. huoshanense*, 47 were significantly changed by exposure to Fe_3_O_4_ NPs based on VIP score from the PLS-DA model and the *P*-value for student's *t*-test.

### Perturbed biological pathways in *D. huoshanense*

Results for the biological pathway analysis showed that 200 mg/L Fe_3_O_4_ NPs induced the perturbation of 8 of 42 biological pathways (*p* < 0.05), including those for pyrimidine metabolism, tyrosine metabolism, beta-alanine metabolism, lysine degradation, alanine, aspartate and glutamate metabolism, pantothenate and CoA biosynthesis, butanoate metabolism, propanoate metabolism (Supplementary Figure 5D). Among them, the first five perturbed pathways were related to nitrogen metabolism. Pantothenate (vitamin B5) was the universal precursor for coenzyme A (CoA); it was also a cofactor in energy yielding reactions, including fatty acid synthesis and carbohydrate metabolism (48). Hence, changes to pantothenate and CoA biosynthesis will inevitably impact downstream carbohydrate and fatty acid metabolism in *D. huoshanense*. Butanoate metabolism and propanoate metabolism were also formed of carbohydrate metabolism. Nitrogen metabolism and carbon metabolism may be the most vulnerable pathways of exposing to Fe_3_O_4_ NPs. Both photosynthetic carbon metabolism and NO${}_{2}^{-}$assimilation occur in chloroplasts, and carbon and nitrogen metabolism require the consumption of organic carbon and energy from CO_2_ assimilation and photosynthesis as well as other electron transport chains. However, in this experiment, Fe_3_O_4_ NPs had a greater effect on chlorophyll in leaves of *D. huoshanense*, which well-explained why carbon and nitrogen metabolism was the most sensitive pathway for NPs exposure.

### Metabolic changes in *D. huoshanense*

A heatmap using 47 differential metabolites revealed significant changes between the treatment group and the control (Supplementary Figure 6). From this, it can be concluded that a marked change in metabolites' content occurred when *D. huoshanense* was hydroponically grown in Fe_3_O_4_ NPs-containing nutrient solution. According to different metabolic functions and pathways, 47 metabolites were divided into six main categories, including carbon metabolism, amino acids and derivatives, antioxidants, fatty acids, signaling molecule, and plant hormone. These compounds will be discussed in the following paragraph on the basis of their own category.

#### Tricarboxylic acid cycle and carbohydrate (carbon metabolism)

**Figure 4:**
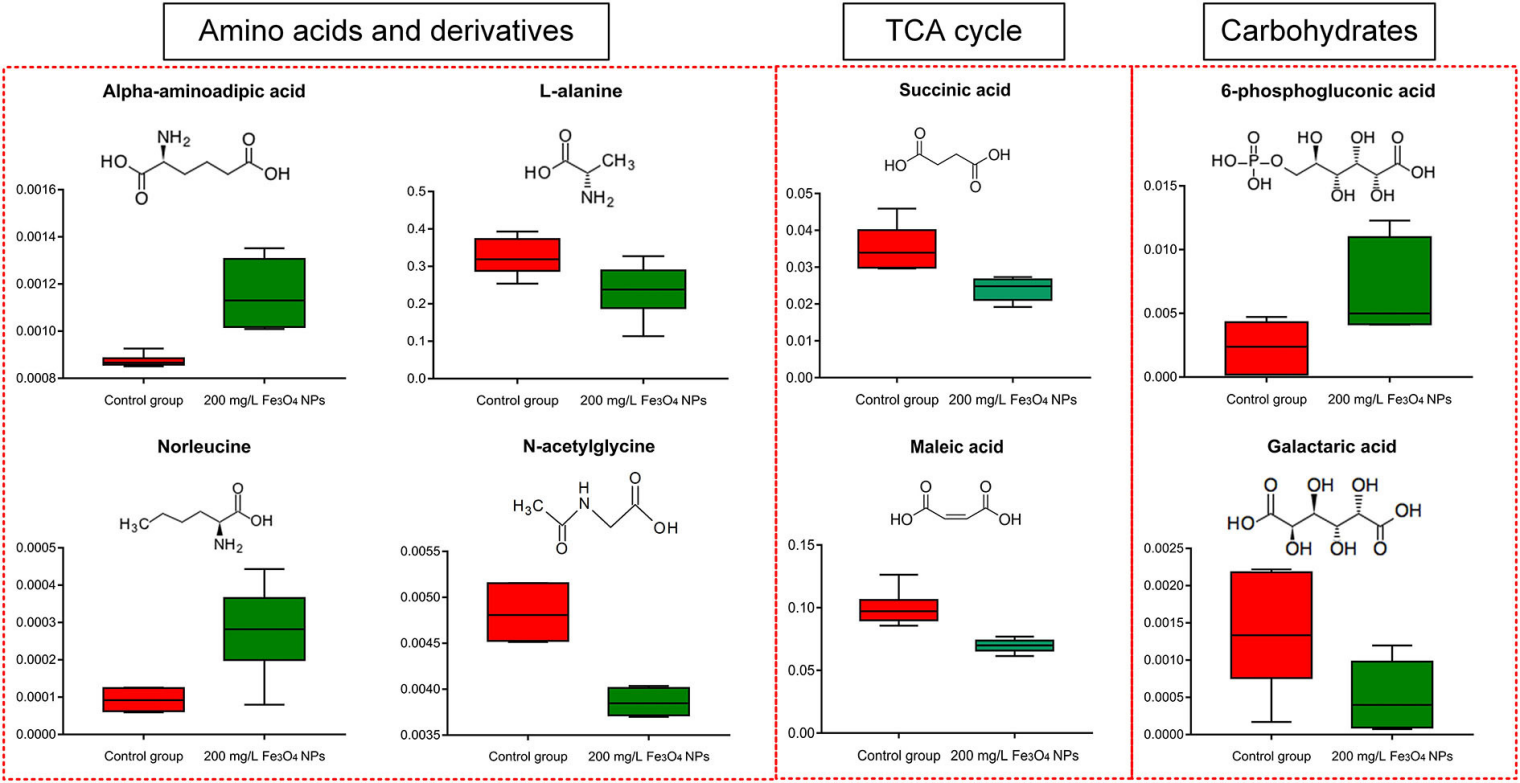
Box plot of relative abundance of amino acids, TCA cycle, and carbohydrates in 2-years-old *D. huoshanense* in 200 mg/L Fe_3_O_4_ NPs and control group (*n* = 6). The Y-axis indicates absolute signal from GC-MS.

Succinic acid, a primary intermediate of the tricarboxylic acid (TCA) cycle, was significantly reduced under Fe_3_O_4_ NPs exposure. The mitochondrial TCA cycle was one of the major pathways of respiration in living organisms. Maleic acid, another TCA cycle intermediate, was also reduced after exposure to Fe_3_O_4_ NPs (Figure 4). The core of cellular respiratory machinery was the TCA cycle. Down-regulation of the TCA cycle's intermediates may indicate the limited respiration in *D. huoshanense*. Enrichment analysis of metabolic pathways also confirmed that the TCA cycle was significantly affected by the exposure to 200 mg/L Fe_3_O_4_ NPs (Supplementary Figure 5D). Photosynthesis and respiration are highly entwined in plant cells, sharing carbon dioxide and oxygen as the product and substrate, respectively (49). Combined with physiological results, 200 mg/L Fe_3_O_4_ NPs increased chlorophyll content and promoted photosynthesis in *D. huoshanense*, while metabolome results showed that Fe_3_O_4_ NPs inhibited plant respiration, reduced organic matter consumption, increased carbohydrate accumulation, and promoted plant growth, which echoed the results of increased polysaccharide content in *D. huoshanense*.

The pentose phosphate pathway (PPP), a way of oxidative decomposition of glucose in plants, was an important pathway. A key intermediate was 6-phosphogluconate, formed by the oxidation of glucose-6-phosphate dehydrogenase (G6PD, EC: 1.1.1.49) that is subsequently oxidatively decarboxylated into ribulose 5-phosphate by 6-phosphogluconate dehydrogenase (6PGD, EC1.1.1.44). Both G6PD and 6pGD were considered rate-limiting enzymes of PPP because their reactions were irreversible in organisms (50). In our study, the Fe_3_O_4_ NPs treatment significantly increased content of 6-phosphogluconate. Combined with the results that the accumulation of glucose in the growth of Fe_3_O_4_ NPs hydroponically cultured *D. huoshanense* was higher than that in the control group, we concluded that Fe_3_O_4_ NPs inhibited the downstream enzyme activity of 6PGD in the PPP pathway of *D. huoshanense*, resulting in the accumulation of the intermediate product (6-phosphogluconate). Several reports demonstrated G6PD activity in plants could be modulated by some metals (51), and it may also act in cell division and salt press (50). In summary, the application of Fe_3_O_4_ NPs may modulate the activity of key enzymes in PPP, thereby changing its 6-phosphogluconic acid content and improving the stress resistance of *D. huoshanense* to better adapt to the environment. Galactoic acid, the main component of pectin, was a dicarboxylic acid produced by the oxidation of D-galacturonic acid (52). Exposing *D. huoshanense* to 200 mg/L Fe_3_O_4_ NPs significantly reduced their galactoic acid content. Sugar acids containing carboxyl and hydroxyl groups can chelate metal ions through multiple binding modes. At physiological pH, galactaric acid coordinates Fe^3+^ through carboxylic oxygen and deprotonated α-hydroxylic group (53). Therefore, the decreased galactoic acid content may be closely related to the increased Fe^3+^ content of *D. huoshanense*. Both the TCA and carbohydrates were important components of carbon metabolism. In conclusion, Fe_3_O_4_ NPs hydroponics exerted a certain influence on carbon metabolism of *D. huoshanense* plants.

#### Amino acids (nitrogen metabolism) and derivatives

Amino acids are essential components of primary metabolism in plants. They not only constitute proteins, but also figure prominently in many physiological processes, by acting as osmolytes, regulating ion transport, contributing to redox-homeostasis, participating in the detoxification of heavy metals, being precursors of plant signaling molecules and defense metabolites (54). Our results showed that most amino acids were influenced by Fe_3_O_4_ NPs negligibly, except α-aminoadipic acid (α-AAA), L-alanine, norleucine, and the amino acid derivative N-acetylglycine (Figure 4). In plant tissues, α-aminoadipic acid is obtained from the catabolism of lysine via the saccharopine pathway (55). The level of α-AAA was promoted significantly in *D. huoshanense* based on its exposing to Fe_3_O_4_ NPs, perhaps because of its response to oxidative stress. Similar to our results, Moulin et al. found that the saccharopine pathway was associated with the stress response in rapeseed (56). Norleucine is generated by the leucine biosynthetic pathway with pyruvate or α-ketobutyric acid replacing α-ketoisovaleric acid as the initial substrate (57). The norleucine content was also significantly increased upon exposure to Fe_3_O_4_ NPs, whereas the contents of L-alanine and N-acetylglycine both decreased. The TCA cycle can produce abundant energy and small molecule precursors, providing the basis for downstream metabolism, for example, intermediates during carbon assimilation and oxidation provide carbon shelves for nitrogen assimilation. hence, downregulation of the TCA cycle could lead to a substantial reduction in downstream response metabolites, such as purine metabolite (adenine, deoxyadenosine, 1-methyladenosine) and pyrimidine metabolite (3-hydroxypropionic acid, cytidine, thymidine, thymine, uracil).

#### Antioxidants

**Figure 5:**
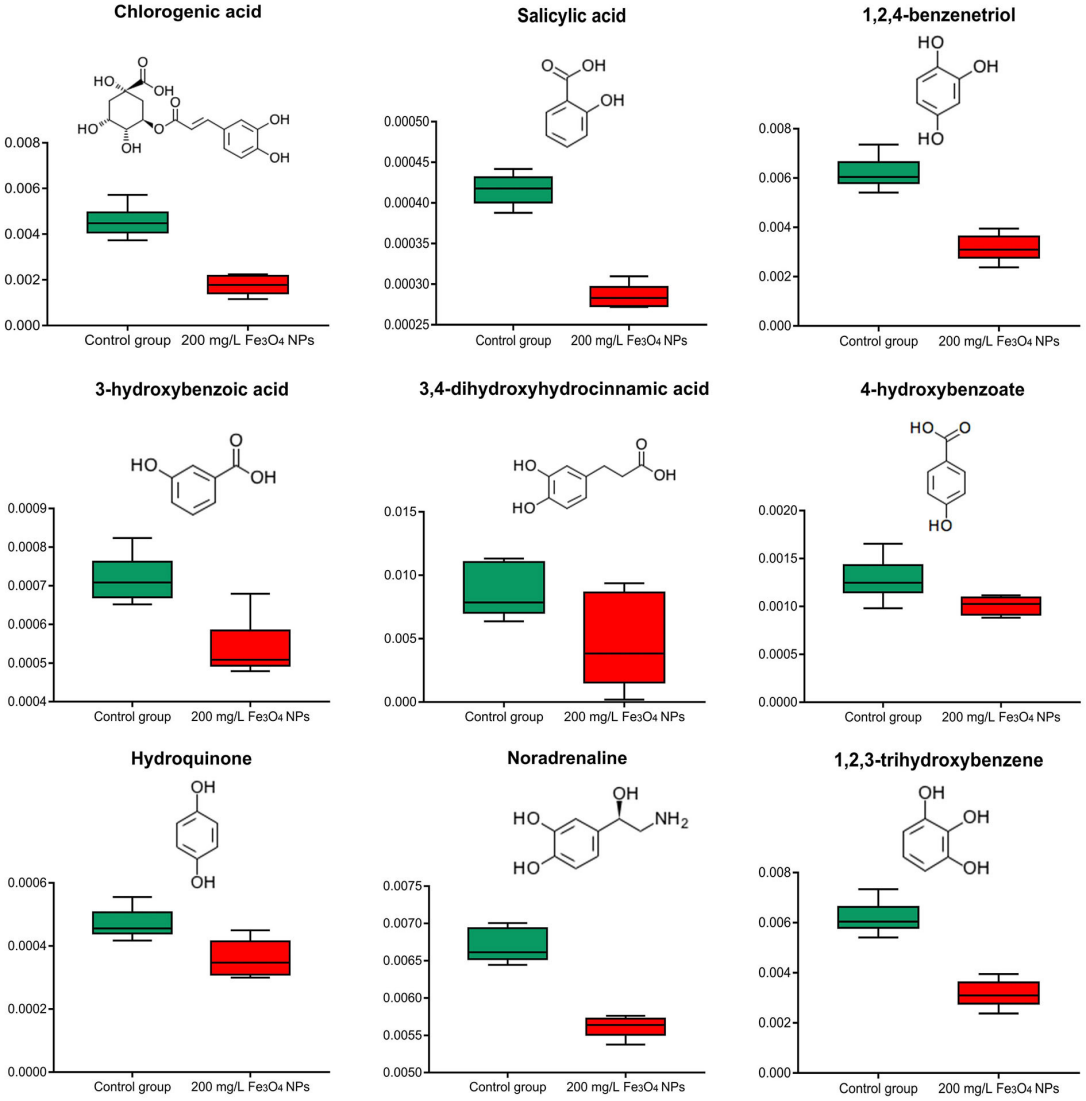
Box plot of relative abundance of Antioxidants in 2-years-old *D. huoshanense* in 200 mg/L Fe_3_O_4_ NPs and control group (*n* = 6). The Y-axis indicates absolute signal from GC-MS.

Non-enzymatic antioxidants in plants include ascorbic acid (ASA), glutathione, tocopherol, and phenolic compounds. Compared with the control, the contents of ASA and tocopherol in the Fe_3_O_4_ NPs treatment group were not significantly changed. Based on the available genetic evidence, chlorophyll degradation and sequential phytol phosphorylation yield phytyl diphosphate (PDP), which is the pentenyl precursor for tocopherol biosynthesis (58). The fact that tocopherol content did not increase in *D. huoshanense* with 200 mg/L Fe_3_O_4_ NPs suggested their degradation was not accelerated, which allowed chlorophyll to accumulate. Chlorogenic acid is one of the most widespread soluble phenolic chemicals in plants, as a byproduct of phenylpropanoid pathway acid pathway and an important precursor of flavonoid biosynthesis (59). The Chlorogenic acid content was significantly reduced in the treatment group; moreover, a number of metabolites with the capacities of ROS scavenging, namely 4-hydroxybenzoate, 3-hydroxybenzoic acid, 3, 4-dihydroxyhydrocinnamic acid, salicylic acid, hydroquinone, noradrenaline, 1, 2, 3-trihydroxybenzene, 1, 2, 4-benzenetriol were decreased by 16–49% in response to the Fe_3_O_4_ NPs compared with the control (Figure 5). On the one hand, these substances are consumed as reducing agents, acting as hydrogen donors to inhibit or quench a free radical (60). Given the large amounts of ferric and ferrous iron available on the surface of Fe_3_O_4_ NPs, they exhibit peroxidase-like activity because of their ability to catalyze the oxidation of peroxidase substrates (61). We suggest that the POD mimetic activity exerted by Fe_3_O_4_ NPs scavenges some ROS in treated plants thereby protecting the endogenous non-enzymatic antioxidant system of *D. huoshanense*. Additionally, there was abundant evidence that phenolic substances and polyphenol oxidase (PPO) activities temporally declineed during flower development and fruit ripening (62, 63). We found that the flowering of *D. huoshanense* could be hastened by the iron treatment, which might also be a reason why the polyphenolic content was reduced in the treatment group. In sum, the changes in antioxidant-related compounds indicated that Fe_3_O_4_ NPs may modulate antioxidant defense pathways in *D. huoshanense*.

#### Fatty acids

**Figure 6:**
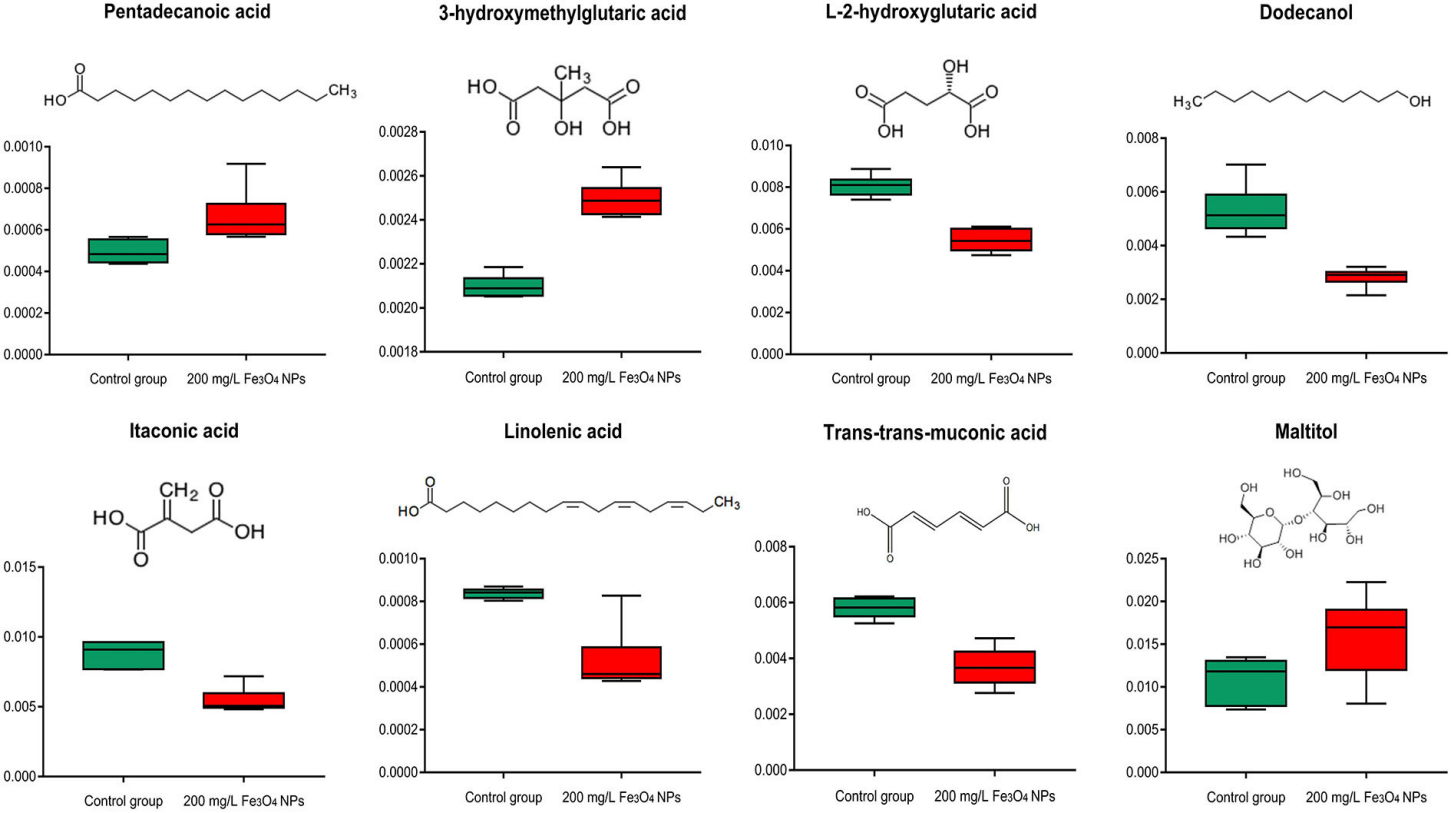
Box plot of relative abundance of fatty acids in 2-years-old *D. huoshanense* in 200 mg/L Fe_3_O_4_ NPs and control group (*n* = 6). The Y-axis indicates absolute signal from GC-MS.

Not only served as the major source of stored energy, fatty acids also constitute an essential component of cellular membranes and are involved in stress responsive activities (64). Fatty acids induce stress resistance in plants through remodeling cell membrane fluidity (65) and work as modulators of defense gene expression (66). Elaidic acid, an unsaturated fatty acid, was trans-isomeric of oleic acid and reported as a membrane component (Supplementary Figure 7). Pentadecanoic acid, a saturated phospholipid fatty acid, was an essential component of the phospholipid bilayer. In this study, both pentadecanoic acid and elaidic acid were significantly increased in *D. huoshanense* exposed to Fe_3_O_4_ NPs (Figure 6), perhaps as an adaptation of this plant to environmental changes. Another significantly up-regulated one was 3-hydroxymethylglutaric acid; in contrast, L-2-hydroxyglutaric acid and several unsaturated fatty acids (itaconic acid, trans-trans-muconic acid and linolenic acid) were down-regulated significantly. In addition, unsaturated fatty alcohol dodecanol was also significantly reduced whereas the content of fatty acyl glycosides (maltitol) was significantly increased. All these metabolite changes may indicate Fe-induced reprogramming of lipid membrane composition of *D. huoshanense*. Lipid peroxidation caused by unsaturated fatty acids is a chain reaction, which can influence free radical in cell membranes. Clearly, a potential reason for the observed changes of fatty acids is lipid peroxidation. It might also because that *D. huoshanense* regulates the fluidity of cell membranes in roots and restricts the infiltration of excess iron ions into its cells, thereby preventing excess iron from damaging the plant.

#### Signaling molecule/plant hormone

Salicylic acid (SA) not only acts as a phenolic antioxidant but also as a pivotal plant hormone that takes part in multiple plant physiological processes, including immune responses, modulation of opening and closing of stomatal aperture, seedling germination, and photosynthesis (67). SA can act as a signaling molecule that coordinates effective defense responses by activating defense gene expression (68). Strangely, unlike most plants under oxidative stress, the content of SA in *D. huoshanense* significantly decreased under Fe_3_O_4_ NPs exposure. The effect of filar spraying Fe_3_O_4_ NPs on tobacco leaves was studied for their resistance to tobacco mosaic virus (TMV) (69). They found that Fe_3_O_4_ NPs entered leaf cells, then transferred and accumulated in the full body of *Nicotiana benthamiana*, coupled with increasing SA biosynthesis and expression levels of SA-responsive PR genes (PR1 and PR2), thereby enhancing resistance of *N. benthamiana* against TMV.

Research has shown that Fe_3_O_4_ NPs can selectively adsorb phytochemicals containing carboxyl groups or o-phenolic hydroxyl groups, and Fe(III) ions may bind more strongly to phytochemicals containing a SA moiety (70). Similar study of different iron-based nanomaterials on the physiological effects of rice, found that a low dose (50 mg/L) Fe_3_O_4_ NPs could reduce oxidative press and decrease the contents of stress-related phytohormones, such as indole-3-acetic acid and gibberellin (71). Taken together, the down regulation of SA may represent an adaptation of *D. huoshanense* to Fe_3_O_4_ NPs exposure.

#### Other differential metabolites

Cholesterol is also present in plant cells although is generally below the 1% of total sterols in plants (72). In plants, cholesterol is the precursor of many metabolites with biological activities (73). In addition, it is a component of cell membranes and leaf surface lipids (74). The Fe_3_O_4_ NPs treatment reduced the cholesterol content in *D. huoshanense* plants, perhaps because cholesterol was used to regulate the fluidity of cell membrane phospholipid molecules. Finally, some substances not annotated in the KEGG database also underwent significant changes in response to Fe_3_O_4_ NPs treatment, including glucosaminic acid, 3,6-anhydro-d-galactose, xylofuranose, citrazinic, beta-gentiobiose, 1-butylamine, isobutene glycol, L-alanine-alanine, 3-dehydroshikimic acid.

## Conclusion

In this study, we investigated the response of Fe_3_O_4_ NPs to *D. huoshanense* by hydroponics. Our data showed that Fe_3_O_4_ NPs at 100 and 200 mg/L had no significant toxic effect on *D. huoshanense*. In contrast, exposure to Fe_3_O_4_ NPs increased leaf chlorophyll content in *D. huoshanense*. In addition, the content of major bioactive substances (polysaccharides) was also increased to different extents, and according to ICP-MS metallomics results, Fe_3_O_4_ NPs hydroponics significantly increased the absorption and accumulation of Fe and other trace elements (Mn, Co, B, Mo) in *D. huoshanense*, and GC-MS-based metabolomics showed that *D. huoshanense* underwent certain metabolite reprogramming responses to Fe_3_O_4_ NPs, benefit from the antioxidant enzyme mimicking activities of Fe_3_O_4_ NPs, it replaces part of the functions of natural small molecule antioxidants in *D. huoshanense*, the decrease of most phenolic metabolites and fatty acids implied the Fe_3_O_4_ NPs altered the lipid membrane of *D. huoshanense* cells, but did not require the accumulation of phenols to enhance their antioxidant defense system to better cope with stress. In addition, Fe_3_O_4_ NPs also interfered with amino acid metabolic pathways, which suggested they can alter the dynamics of nitrogen metabolism and reallocation of energy. These results indicate that Fe_3_O_4_ NPs have potential applications as nano-fertilizers on *D. huoshanense*, which is a reference for making some nutrients that are not easily absorbed by plants into nanomaterials to improve their utilization. In this study, the contribution of ferric iron, divalent iron, and NPs in Fe_3_O_4_ NPs to metabolite changes is still unclear, and future work should address the effects of nanoparticles with different valence states iron ions on polysaccharides (molecular weight, glycosidic bonds, and spatial structure) and metabolomics in *D. huoshanense* at the same level of bioavailability, their benefits or toxicity to *D. huoshanense* should be interpreted with corresponding nanomaterials with larger concentration gradients.

## Data availability statement

The data presented in the study are deposited in the MetaboLights repository, https://www.ebi.ac.uk/metabolights/, accession number MTBLS5735.

## Author contributions

ZW: conceptualization, methodology, and writing—original draft. JW: software and investigation. ZS: funding acquisition. WJ: methodology. YL: resources. JT: resources and visualization. XM: formal analysis. XS: software and validation. LWu: software and methodology. LWa: visualization and data curation. XG: validation. DP: project administration and funding acquisition. SX: conceptualization, methodology, supervision, and writing—review and editing. All authors contributed to the article and approved the submitted version.

## Funding

This work was supported by National Natural Science Foundation of China (No. U19A2009), Open Project of Provincial and Ministerial Scientific Research Platform, Fuyang Normal University (No. FSKFKT010D), Natural Science Foundation of Anhui Province (No. 1908085MH268), Key Natural Science Research Projects in Anhui Universities (No. KJ2019A0453 and KJ2018A0275), Key Natural Science Research Projects in Anhui Universities (No. KJ2021A0676), and Anhui University Collaborative Innovation Project (No. GXXT-2019-043 and No. GXXT-2019-049).

## Conflict of interest

The authors declare that the research was conducted in the absence of any commercial or financial relationships that could be construed as a potential conflict of interest.

## Publisher's note

All claims expressed in this article are solely those of the authors and do not necessarily represent those of their affiliated organizations, or those of the publisher, the editors and the reviewers. Any product that may be evaluated in this article, or claim that may be made by its manufacturer, is not guaranteed or endorsed by the publisher.

## Supplementary material

Supplementary Figure 1 TEM image and size distribution of Fe_3_O_4_ NPs.

Supplementary Figure 2 Physiological indexes detection after treatment. Stem biomass **(A)**, the number of flowering plants **(B)** and time to flower **(C)** of *D. huoshanense* exposed to different doses of Fe_3_O_4_ NPs (0, 100, and 200 mg/L) for 21 days. Chlorophyll a **(D)**, chlorophyll b **(E)** and carotenoid content **(F)** of *D. huoshanense* leaves exposed to different doses of Fe_3_O_4_ NPs (0, 100, and 200 mg/L) at different time (0, 7, 14, and 21 days). Data are means of three replicates. FW represents the fresh weight of the samples. Error bars represent standard deviation. Different letters stand for statistical differences at *p* < 0.05.

Supplementary Figure 3 Growth records of *D. huoshanense* in nutrient solution with different doses of Fe_3_O_4_ NPs (0, 100, and 200 mg/L).

Supplementary Figure 4 Total flavonoid content **(A)**, total alkaloid content **(B)** of *D. huoshanense* leaves exposed to different doses of Fe_3_O_4_ NPs (0, 100, and 200 mg/L) at different time (0, 7, 14, and 21 day). Data are means of three replicates. FW represents the fresh weight of the samples. Error bars represent standard deviation. Different letters stand for statistical differences at *p* < 0.05.

Supplementary Figure 5 Principal Component Analysis (PCA) **(A)** and Partial least squares discriminate analysis (PLS-DA) **(B)** The number of differential metabolites between control group and 200 mg/L Fe_3_O_4_ NPs, **(C)** Score plots of metabolic profiles in *D. huoshanense* treated with 200 mg/L Fe_3_O_4_ NPs, **(D)** Top 20 enriched pathways, red line dotted line shows *P*-value is 0.01 and blue dotted line shows *P*-value is 0.05.

Supplementary Figure 6 Heatmap of 47 differential metabolites.

Supplementary Figure 7 Elaidic acid content of *D. huoshanense* in 200 mg/L Fe_3_O_4_ NPs and control group (*n* = 6).
