# Supplementary material for: ICP-MS based metallomics and GC-MS based metabolomics reveals the physiological and metabolic responses of Dendrobium huoshanense plants exposed to Fe3O4 nanoparticles
Source: Front Nutr. 2022 Sep 23;9:1013756. doi: 10.3389/fnut.2022.1013756 (PMC9558897; doi:10.3389/fnut.2022.1013756)

Supplementary Figure 1

TEM image and size distribution of Fe_3O_4 NPs.

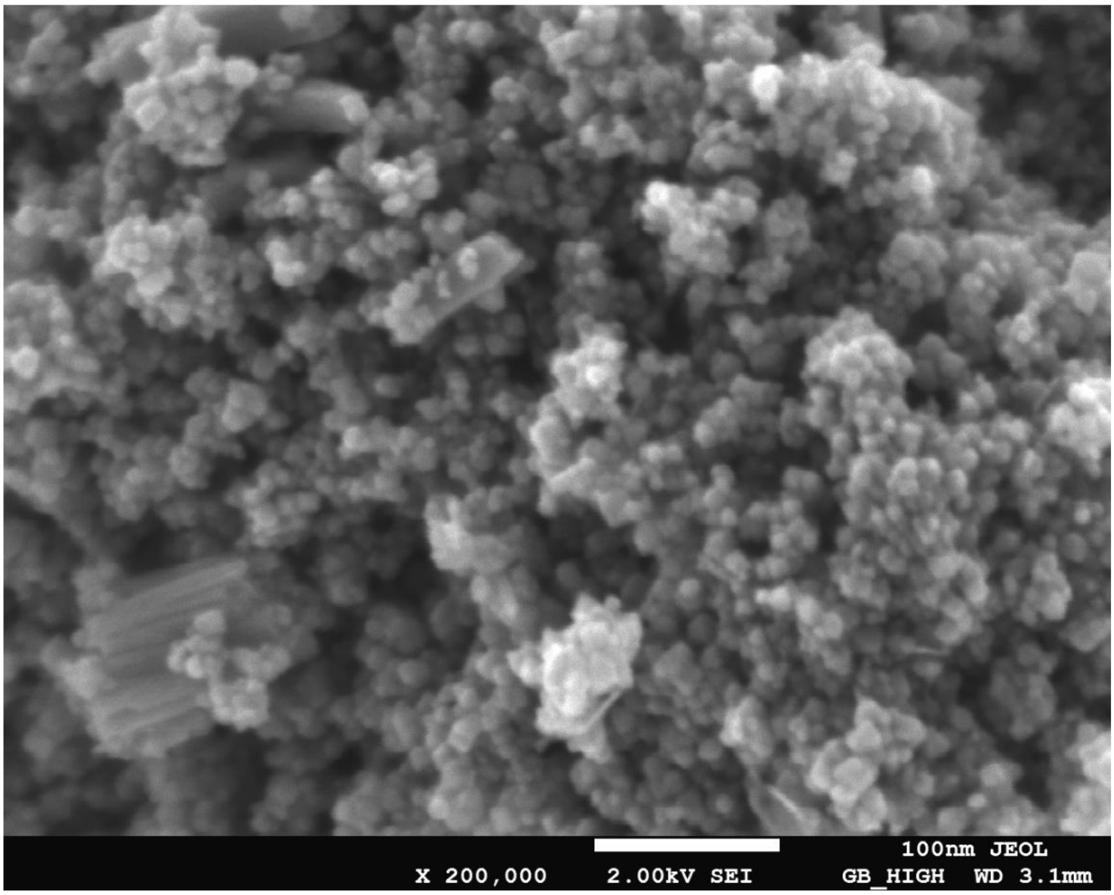

Supplementary Figure 2

Physiological indexes detection after treatment. Stem biomass **(A)**, the number of flowering plants **(B)** and time to flower **(C)** of *D. huoshanense* exposed to different doses of Fe_3O_4 NPs (0, 100 and 200 mg/L) for 21 days. Chlorophyll a **(D)**, chlorophyll b **(E)** and carotenoid content **(F)** of *D. huoshanense* leaves exposed to different doses of Fe_3O_4 NPs (0, 100 and 200 mg/L) at different time (0, 7, 14 and 21 days). Data are means of three replicates. FW represents the fresh weight of the samples. Error bars represent standard deviation. Different letters stand for statistical differences at $p < 0.05$.

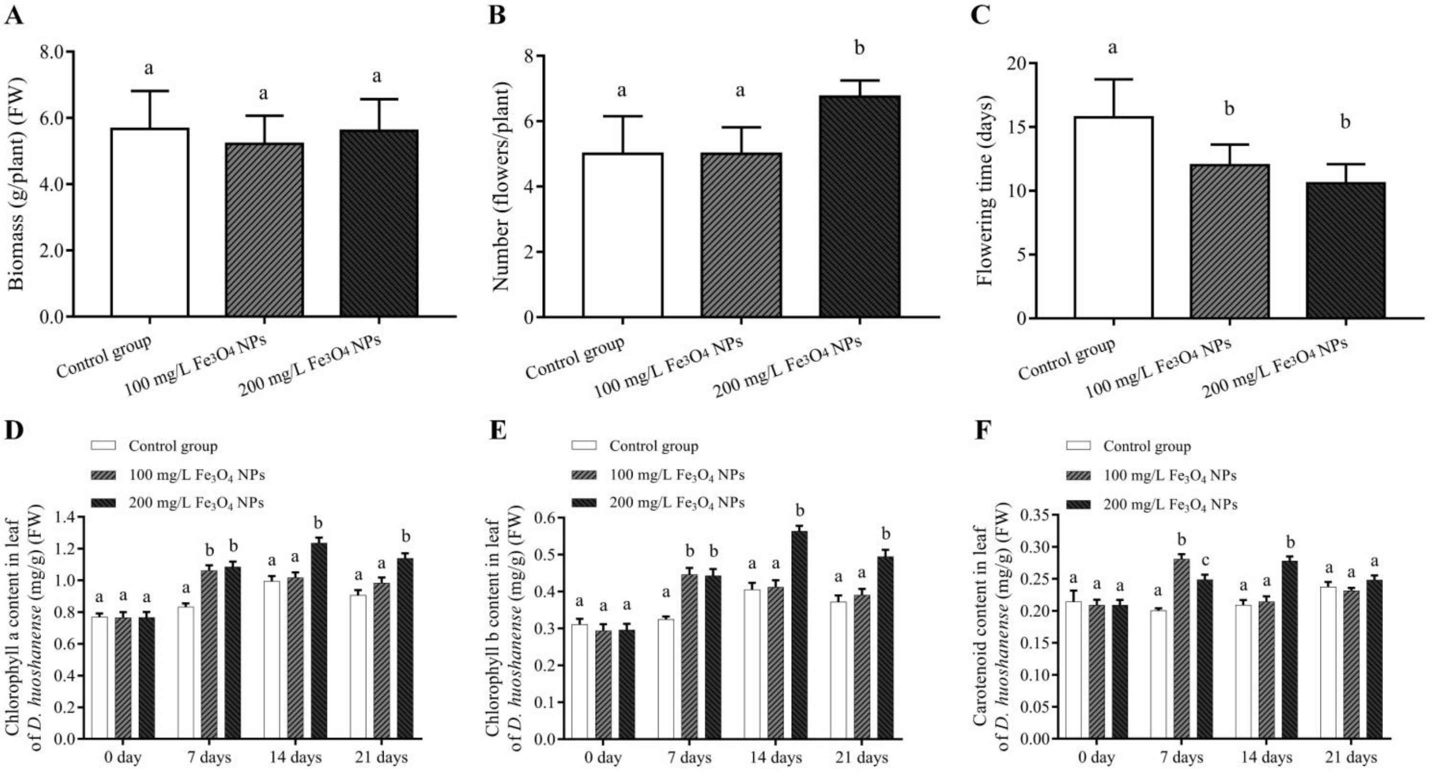

Supplementary Figure 3

Growth records of *D. huoshanense* in nutrient solution with different doses of Fe_3O_4 NPs (0, 100 and 200 mg/L).

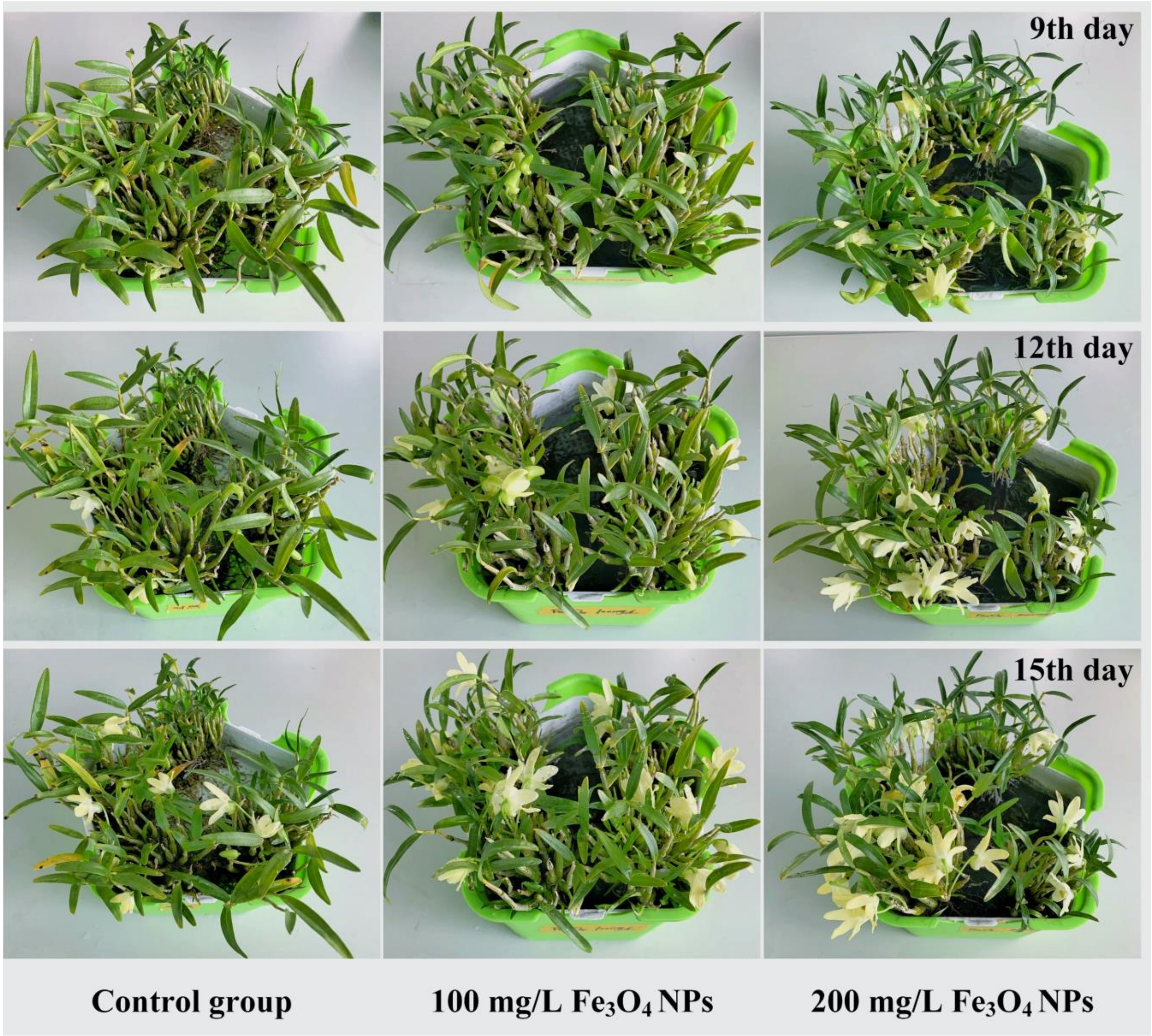

Supplementary Figure 4

Total flavonoid content **(A)**, total alkaloid content **(B)** of *D. huoshanense* leaves exposed to different doses of Fe_3O_4 NPs (0, 100 and 200 mg/L) at different time (0, 7, 14 and 21 day). Data are means of three replicates. FW represents the fresh weight of the samples. Error bars represent standard deviation. Different letters stand for statistical differences at $p < 0.05$.

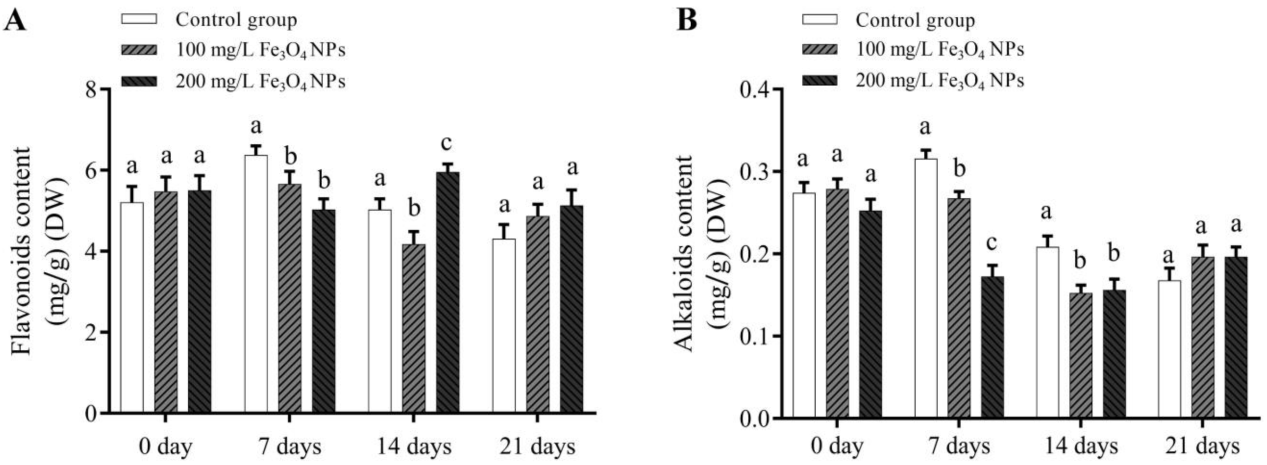

Supplementary Figure 5

Principal Component Analysis (PCA) (**A**) and Partial least squares discriminate analysis (PLS-DA) (**B**) The number of differential metabolites between control group and 200 mg/L Fe_3O_4 NPs, (**C**) Score plots of metabolic profiles in *D. huoshanense* treated with 200 mg/L Fe_3O_4 NPs, (**D**) Top 20 enriched pathways, red line dotted line shows P-value is 0.01 and blue dotted line shows P-value is 0.05.

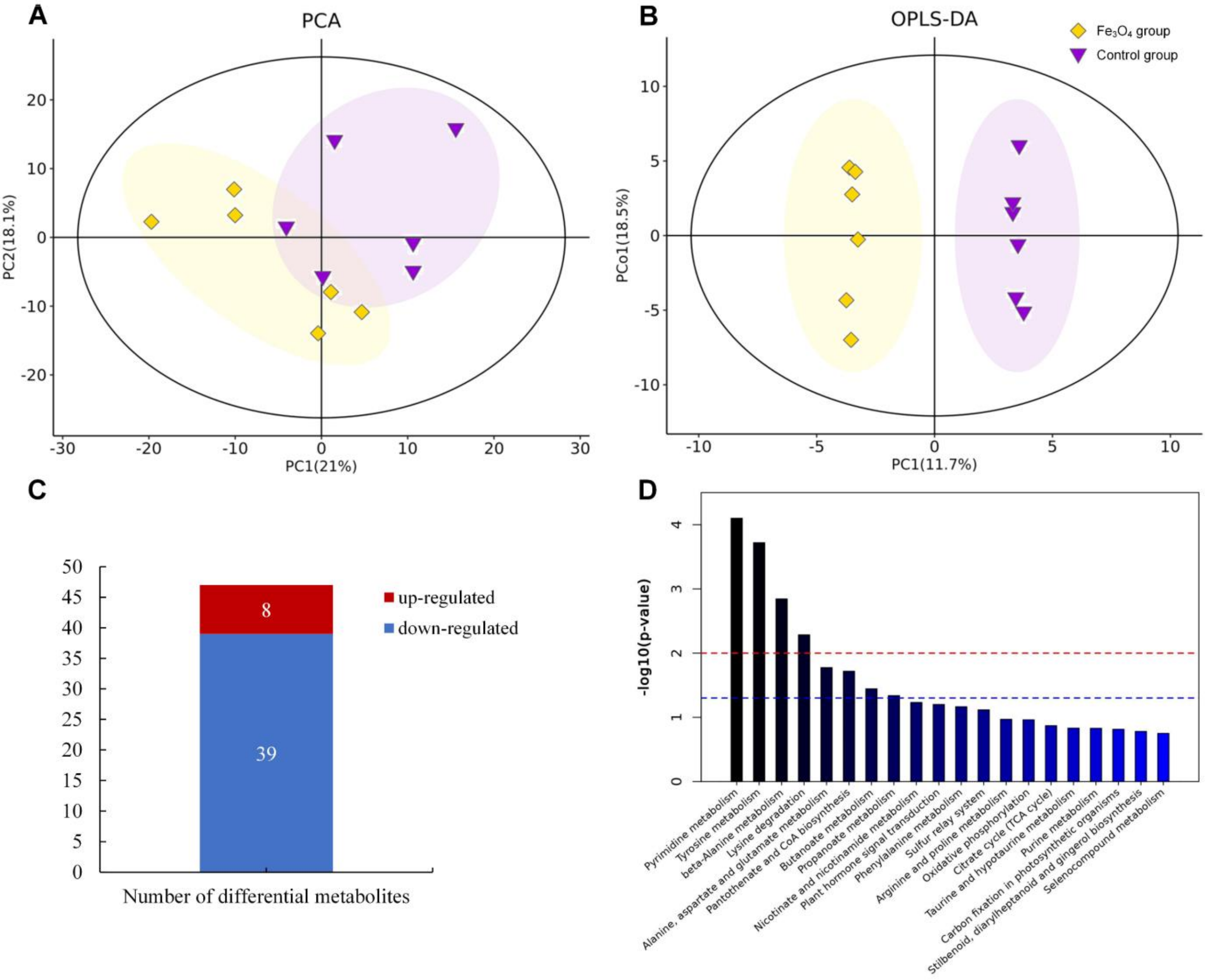

Supplementary Figure 6

Heatmap of 47 differential metabolites.

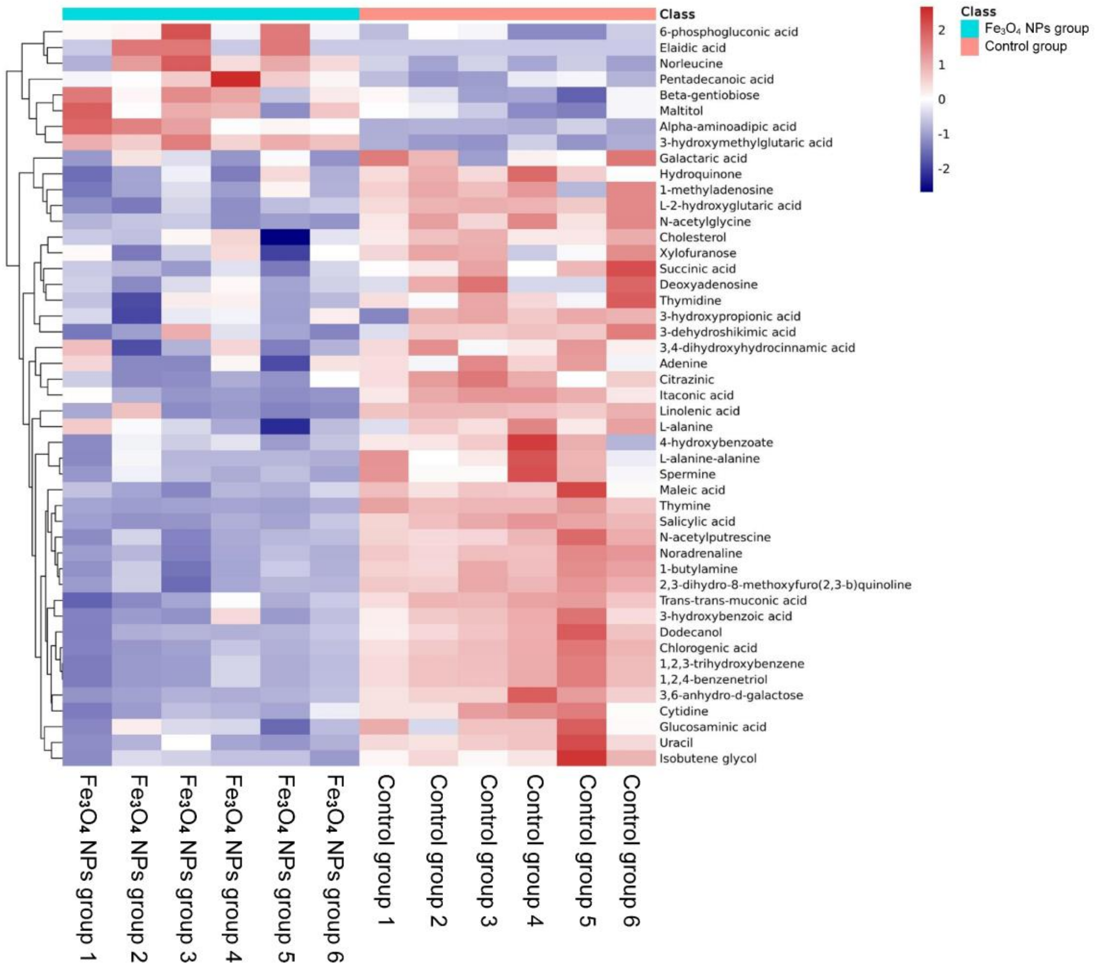

Supplementary Figure 7

Elaidic acid content of *D. huoshanense* in 200 mg/L Fe_3O_4 NPs and control group (n = 6).

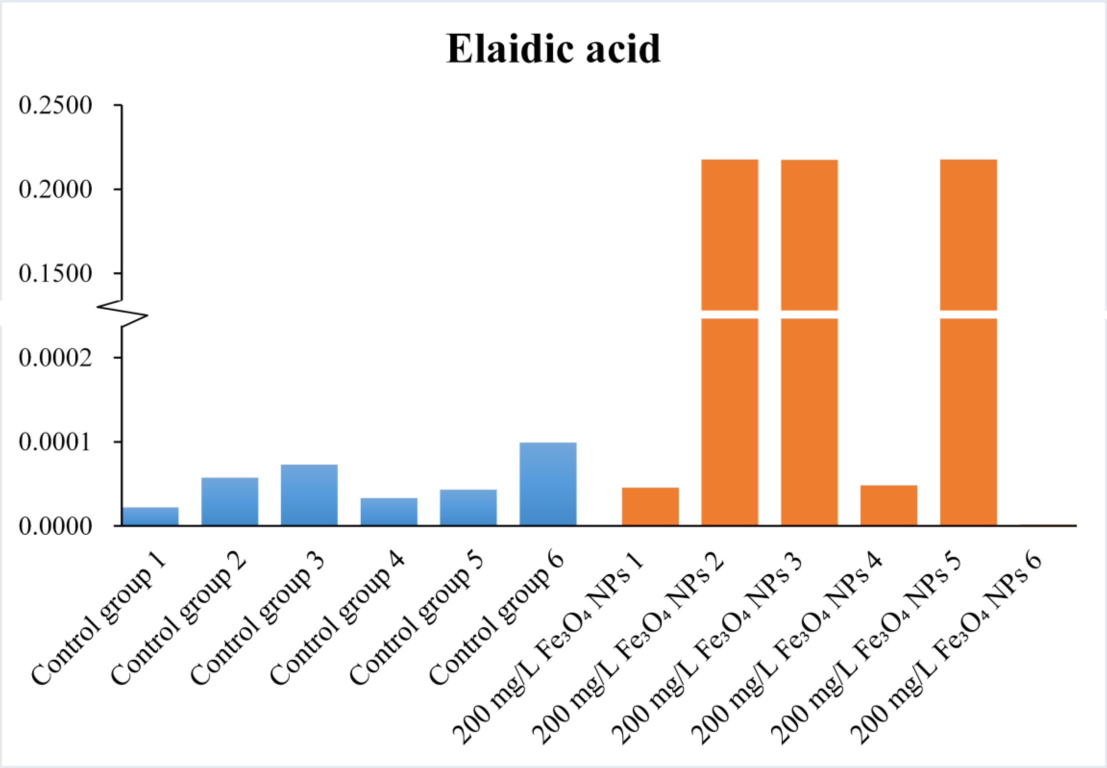

Supplement: Supplementary Figure 1 — TEM image and size distribution of Fe3O4 NPs. [file Data_Sheet_1.pdf]
